# Defining paleoclimatic routes and opportunities for hominin dispersals across Iran

**DOI:** 10.1371/journal.pone.0281872

**Published:** 2023-03-01

**Authors:** Mohammad Javad Shoaee, Paul S. Breeze, Nick A. Drake, Seyyed Milad Hashemi, Hamed Vahdati Nasab, Sebastian F. M. Breitenbach, Thomas Stevens, Nicole Boivin, Michael D. Petraglia

**Affiliations:** 1 Department of Archaeology, Max Planck Institute for Geoanthropology, Jena, Germany; 2 Department of Geography, King’s College London, London, United Kingdom; 3 Department of Archaeology, Faculty of Humanities, Tarbiat Modares University, Tehran, Iran; 4 Department of Geography and Environmental Sciences, Northumbria University, Newcastle upon Tyne, United Kingdom; 5 Department of Earth Sciences, Uppsala University, Uppsala, Sweden; 6 School of Social Science, the University of Queensland, Brisbane, Queensland, Australia; 7 Department of Anthropology and Archaeology, University of Calgary, Calgary, Alberta, Canada; 8 Department of Anthropology, National Museum of Natural History, Smithsonian Institution, Washington, DC, United States of America; 9 Human Origins Program, National Museum of Natural History, Smithsonian Institution, Washington, DC, United States of America; 10 Australian Research Centre for Human Evolution, Griffith University, Griffith, Australia; New York University, UNITED STATES

## Abstract

Fossil and archaeological evidence indicates that hominin dispersals into Southwest Asia occurred throughout the Pleistocene, including the expansion of *Homo sapiens* populations out of Africa. While there is evidence for hominin occupations in the Pleistocene in Iran, as evidenced by the presence of Lower to Upper Paleolithic archaeological sites, the extent to which humid periods facilitated population expansions into western Asia has remained unclear. To test the role of humid periods on hominin dispersals here we assess Paleolithic site distributions and paleoenvironmental records across Iran. We developed the first spatially comprehensive, high-resolution paleohydrological model for Iran in order to assess water availability and its influence on hominin dispersals. We highlight environmentally mediated routes which likely played a key role in Late Pleistocene hominin dispersals, including the expansion of *H*. *sapiens* and Neanderthals eastwards into Asia. Our combined analyses indicate that, during MIS 5, there were opportunities for hominins to traverse a northern route through the Alborz and Kopet Dagh Mountains and the Dasht-I Kavir desert owing to the presence of activated fresh water sources. We recognize a new southern route along the Zagros Mountains and extending eastwards towards Pakistan and Afghanistan. We find evidence for a potential northern route during MIS 3, which would have permitted hominin movements and species interactions in Southwest Asia. Between humid periods, these interconnections would have waned, isolating populations in the Zagros and Alborz Mountains, where hominins may have continued to have had access to water.

## Introduction

Despite relatively little archaeological field research across Iran, a number of Lower to Upper Paleolithic sites have been identified. The very first Paleolithic finds in the Iranian Plateau were reported by geologists conducting field surveys in the early nineteenth century [1, 2], followed by occasional excavations in the region up until the 1950s (e.g., [3–6]), when the Zagros was thrust into the limelight by the discovery of Neanderthal fossils [6, 7]. These fossil finds triggered intense interest from international scholars who focused on the Zagros Mountains. Unfortunately, the majority of these excavations were only briefly reported and frequently defined by imprecise excavation methods, with doubts about dating and site formation remaining to this day (e.g., [7–11]). From the 1970s onward, political events inhibited regional archaeological research, and intensive work has only renewed over the last two decades, when many excavations were initiated utilizing multidisciplinary scientific approaches. These Paleolithic site excavations were mainly focused on caves and rock shelters in the Zagros Mountains, with investigation of open-air sites largely neglected. However, numerous open-air Paleolithic sites have recently been discovered in many parts of Iran, and increasing attention has begun to be paid to them (e.g., [12–17]). Thus, a growing number of Paleolithic field investigations have been initiated, and the importance of the region in relation to the dispersal of *H*. *sapiens* outside Africa, and our interactions with other hominins in Asia, is becoming increasingly apparent [18, 19].

**Fig 1:**
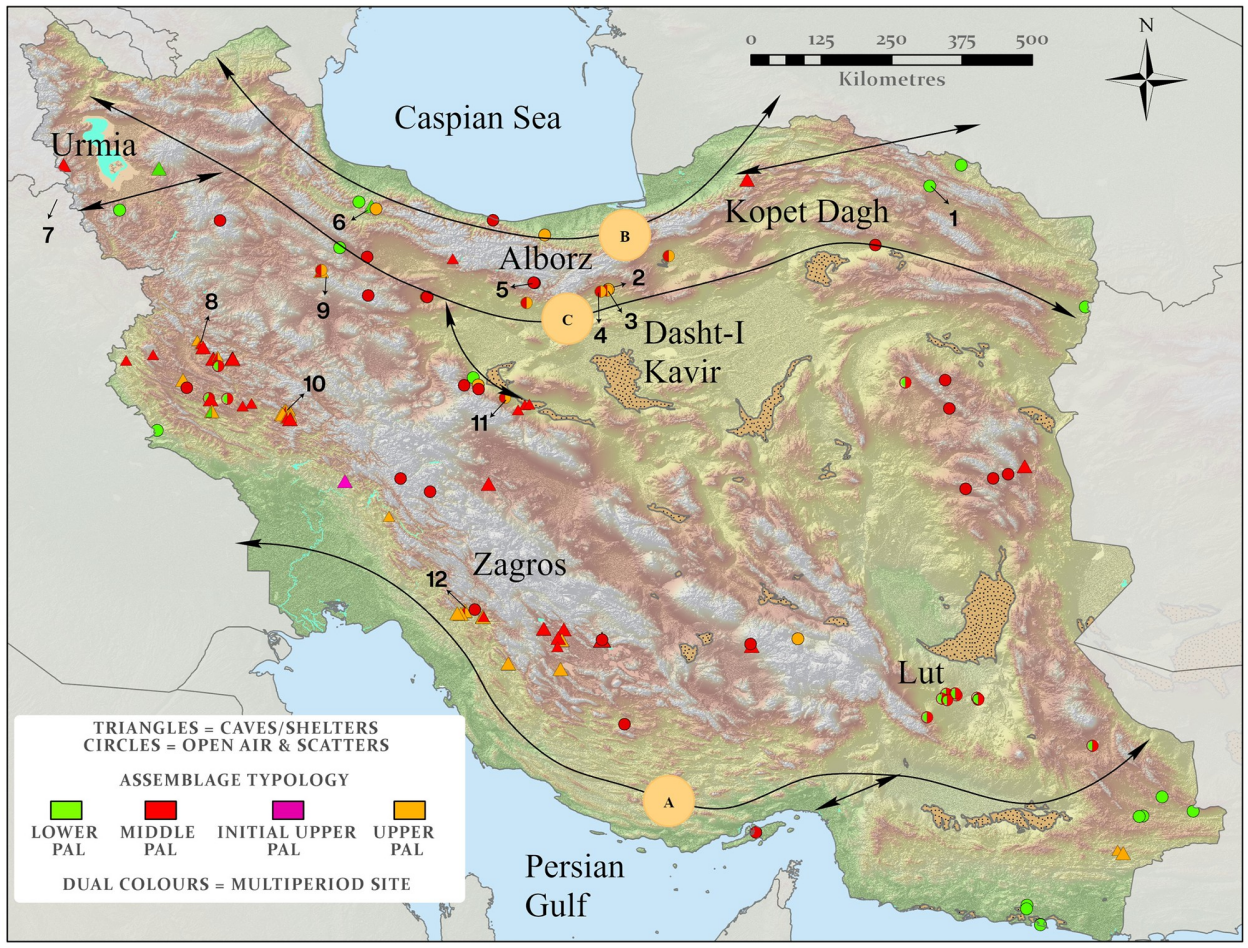
The distribution of key Paleolithic sites in Iran differentiated by lithic typology. The sites are overlain on elevation data (SRTM), progressing from low (green) to high (white) altitudes. The brown speckled polygons mark dune fields. Regions referenced in the text are labelled. Dispersal routes (A-C) previously suggested by Vahdati Nasab et al. [13] are marked as black arrows. The location of archaeological sites discussed in the text is numbered as follow; 1. Kashafrud, 2. Chah-e Jam, 3. Mirak, 4. Soofi Abad, 5. Moghanak and Otchunak, 6. Darband, 7. Shanidar, 8. Bawa Yawan, 9. Qaleh Kurd, 10. Kaldar, 11. Qaleh Gusheh, 12. Ghar-e Boof. Map created by one of our authors (PSB).

The archaeological record of Iran indicates that the earliest occupations occurred during the Lower Paleolithic (LP) on the basis of characteristic artifact forms [18]. Most LP lithic artifacts were retrieved from surface scatters or as single finds. An exception is Darband Cave which has yielded multiple Acheulean artifacts though this deposit has not yet been dated [20]. Characteristic lithic finds such as handaxes, choppers, and cleavers are reported from Ganj Par and Darband, Kashafrud, Amar Merdeg and Cham-e Souran [20–22], and most sites are associated with the Acheulean tradition. The distribution of the finds indicate a widespread dissemination of Acheulean tool users across northern and southern Iran (Fig 1). Though no early hominins have been identified in Iran, fossils representative of *Homo erectus* and *Homo heidelbergensis* have been recovered from nearby regions from Early to Middle Pleistocene contexts [23, 24].

Middle Paleolithic (MP) and Upper Paleolithic (UP) sites are more common and their chronology is better understood. Most dates from stratified MP archaeological sites in Iran range from ~150–42 ka, and have been interpreted as evidence for Neanderthal occupations [25–27]. However, this time window also includes a suggested phase of expansion of *H*. *sapiens* into Asia (~70–40 ka), indicated by genetic studies [28, 29], highlighting Iran to be a territory of interest when considering the potential for interactions and introgression between these hominin species. Marine Isotope Stage (MIS) 5, particularly MIS 5e at the peak of the last interglacial, is also of particular significance when considering the dispersal of *H*. *sapiens* populations. This relatively warmer and wetter period has been highlighted as a key driver of early expansions of our species. At this time the adjacent Levant and Arabia were populated by *H*. *sapiens* populations expanding out of Africa [30] with archaeological evidence suggesting these groups may have reached even further east [31]. Thus, despite a current absence of direct dates for any presence of hominins in Iran in MIS 5, the potential for hominin movements through the region remains of significant research interest, particularly as this may have been a period of enhanced climatic amelioration and penetration of the monsoon into southern Iran, in common with other adjacent regions, such as Arabia. Arabia also provides a cautionary tale that this initial apparent absence of MIS 5 sites may not necessarily reflect a true absence, as no securely dated Paleolithic sites existed in Arabia until 2011, since which time a plethora of such sites have been identified, including several from MIS 5 [30, 32–35] associated with periods of monsoonal amelioration. However, the specifics of any environmentally favorable routes which might have facilitated movements into and through Iran, and thus the potential for populations (of either Neanderthals or *H*. *sapiens*) to expand their ranges or interact during this period remain unclear, though suggestions have been proposed previously [13, 18]. The need for further analyses to resolve climatic routes for dispersal has been noted [36], and our study seeks to address this.

The UP is found in many excavated sites in the Zagros and Alborz regions [18] and their chronological timeframe is better understood than that of earlier periods. Studies suggest that several different, well-developed and localized stone-tool industries were present in the UP over a short period, ranging from ~45 to 40 ka [37–41]. The Initial Upper Paleolithic (IUP) makes it appearance at ~42 ka in the Zagros Mountains [41], where its makers may have coexisted with Neanderthals who survived in the region until ~42 ka [25]. As the IUP is considered a marker of *H*. *sapiens* [42], it may indicate that at ~42 ka, *H*. *sapiens* dispersed into the Neanderthal dominated region of the Zagros, suggesting a complex demography for hominins in a region that we are only just beginning to understand.

Vahdati Nasab and colleagues [13] outlined three principal potential dispersal corridors through the region that could have facilitated the above mentioned hominin occupations, one southern route along the coastal plains of the Persian Gulf and the Makran region (Route A in their study and on Fig 1), another route north of the Alborz Mountains along the margin of the Caspian Sea (Route B), and a third along the southern foothills of the Alborz northern edge of the Dasht-I Kavir (Route C). Comparison of these routes with other dispersal routes, including along the Persian Gulf and those identified in GIS least-cost models, reflect the large potential for inland dispersals, contrasting with coastal route models along the Persian Gulf and Gulf of Oman shorelines [18]. The significance of freshwater and likely water-tethered nature of hominin occupations in Iran has previously been raised [36]. To further explore potential routes of dispersal, here we integrate numerous sources of information, including archaeological data, paleoclimatic proxy data, novel paleohydrological maps, and paleoclimatic modelling for key intervals in the Middle and Late Pleistocene. This allows critical examination of the environmental conditions (and particularly freshwater availability) which influenced these dispersal routes as well as hominin range expansions and contractions over time.

### Study region

**Fig 2:**
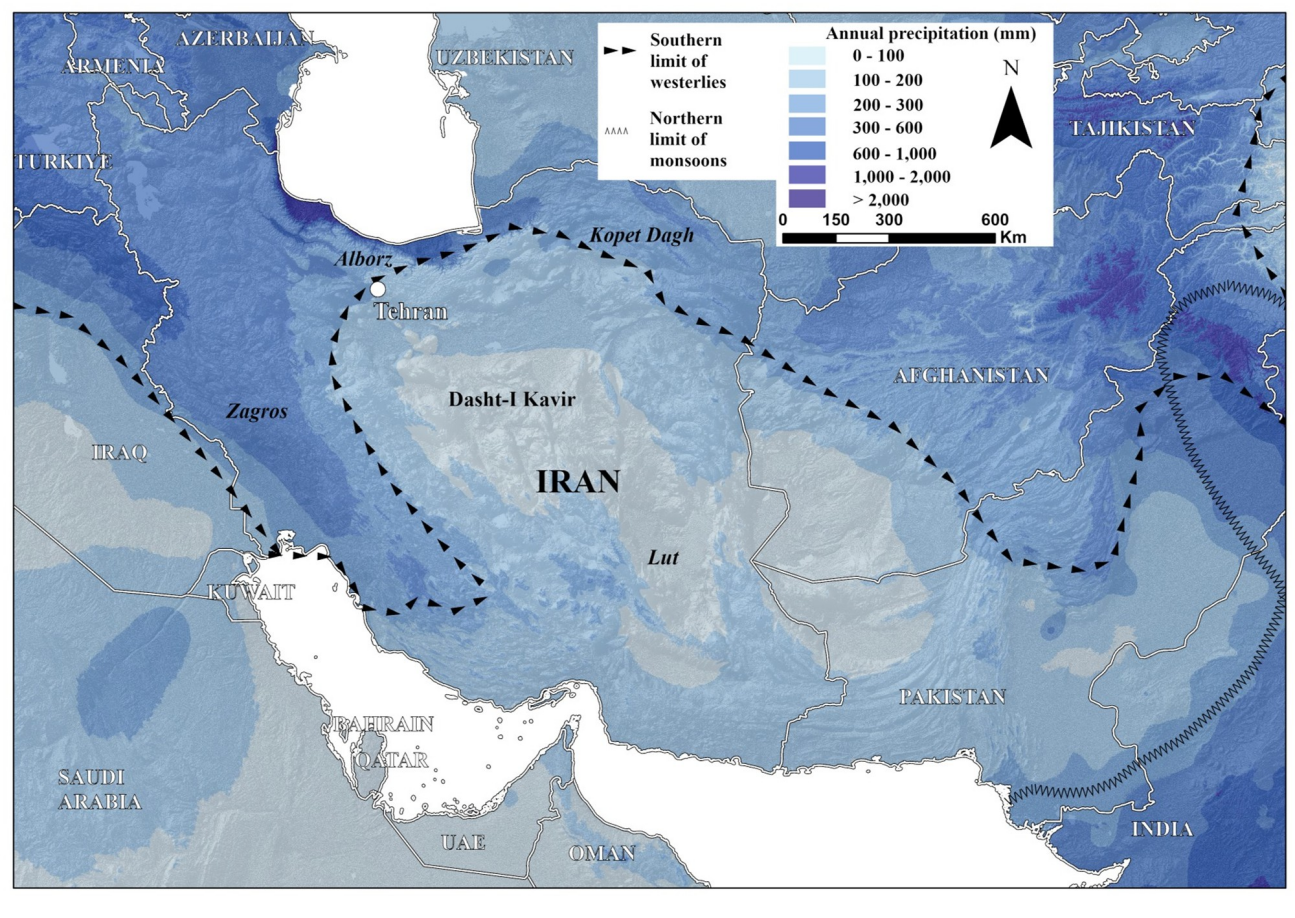
Modern mean annual precipitation for Iran (WorldCLIM). Map depicts the current limits of westerly and monsoonal moisture overlain on monthly rainfall through the year. Monsoon limits come from quantitative analyses of global monsoons [46, 47]. Higher rainfall is found in the north and west, particularly in the Zagros, Alborz and Kopet Dagh Mountains further south rainfall declines in the Dasht-I Kavir and Lut Deserts. Map created by one of our authors (PSB).

Iran is located at the intersection between geographically and archaeologically significant regions of Western Asia. Arabia and the Levant lie to the southwest and the Caucasus Mountains to the northwest, while the Central Asian deserts and steppes are to the northeast, and South Asia to the east. Due to this centricity, hominin interactions or dispersals between these regions of south-west and central Asia would be dependent upon movements through Iran. Iran is physio-geographically heterogeneous (Fig 1), with the Zagros Mountains in the west, the Caspian Sea and the Alborz Mountains to the north, and the Persian Gulf to the south, and two vast deserts, the Dasht-I Kavir and the Lut Desert in its central and southeastern regions. Today, the region is generally characterized by an arid climate (Fig 2), except for the Zagros Mountains, the thin band of high rainfall along the southern Caspian Sea and the northern slopes of the Alborz Mountains, where a lush ecozone known as the Hyrcanian Forest is found, and the semi-arid climate on the Iranian Plateau (Fig 2) [43]. This moisture is mostly provided by mid-latitude westerlies. This landscape heterogeneity would have significantly influenced hominin demography and expansion during the Pleistocene [44, 45].

## Materials and methods

We combined an existing archaeological database with paleoclimate proxy data and combined this information with a novel paleohydrological analyses and the results of paleoclimate modelling to produce an analysis of potential opportunities for Pleistocene dispersals through Iran, as outlined below.

### Archaeological database

In a previous study, we synthesized and assessed 106 Paleolithic sites across the Iranian Plateau exhibiting technotypological attributions ranging from the Lower Paleolithic to the Epipaleolithic [18]. We only included archaeological sites that we considered as possessing reliable chronological ages or clear lithic typologies (Fig 1). Here we further assess the sites presented in Shoaee et al. [18], excluding those from the Epipaleolithic period. Additionally, we added to the database further surface scatters and single finds that have been recorded recently or published in brief reports, bringing our Lower to Upper Paleolithic database to a total of 123 localities across Iran (S1 Table).

### Paleohydrological mapping

To map paleohydrology we used techniques previously established by Breeze et al. [48–50]. Former drainage systems and catchments were mapped using the HydroSHEDS dataset [51] to detect the topographic expression of the most recent configuration of former river channels, and then critiqued and augmented using PALSAR [52] radar data that allows the visual detection of shallow buried channels, and areas of earlier channel switching and drainage capture [53]. A rough determination of the age of the river systems of Iran was established using available data from dating of lava flows intruded along channels [54], and from timescales for the creation of the central Zagros spine basins [55], with results suggesting that in many areas of the country the principal current drainage configurations pre-date the Quaternary [56]. Iran is a tectonically active region, and several studies show evidence for Quaternary tectonic (and salt) deformation in the central plateau [57], with the potential of local alteration of topography and drainage configurations during the time period of interest.

Paleolakes were mapped using multispectral Landsat classification to identify exposed lacustrine deposits [48]. Paleolake deposits were mapped in ENVI by applying the Matched Filtering/Spectral Angle Mapper classification algorithm to a median value mosaic of Landsat Thematic Mapper data acquired between 1984 and 1994 during July to October, processed in and exported from Google Earth Engine. The maximum size of the lakes in each basin was determined by ‘flooding the basin’ to the altitude of the highest detected paleolake deposit using the 90 m SRTM DEM. In this manner, paleolakes and paleochannels were mapped at 90 m spatial resolution across the entire country. To identify any current springs and still extant lakes which may also have served as water sources during the Pleistocene, all locations with a presence of surface water for greater than 90% of the time between 1984 and 2015 were selected from the Pekel et al. [58], Global water occurrence dataset and pre-treated to remove any artificial reservoirs using the GranD dataset [59]. These data were combined and used to calculate distance from water sources to the archaeological sites, to examine the connectivity or otherwise of areas in relatively close proximity to water, and evaluate whether these could have provided routes facilitating dispersals.

To examine the influence of tectonic processes on basin developments we reviewed the geological literature and identified a single reference to the opening of a formerly closed basin that held a Holocene lake at Golbaf, which precluded the ability of our classifications to identify a paleolake at this site. Thus, in this basin, we instead visually identified the former lake extent from DEM and Landsat data and mapped it manually. We also visually scrutinized a Landsat TM band 7,4,1 seamless mosaic for the entire area in order to identify any other large lakes that may have eluded our automated detection due to opening of any previously closed basin. In order to evaluate the spatial distribution of water source availability we calculated the distance from all mapped water sources (paleolake, spring or major drainage system of >1000 km upstream accumulation area) at 90 m spatial resolution using the cumulative cost function in Google Earth Engine.

### Paleoenvironmental proxy data and catchment level analyses

To assess paleoenvironmental dynamics in Iran across the relevant periods, we compiled a spatial database of published continental environmental proxy records. Site locations were derived from the parent publications for these proxy records. We selected paleoenvironmental reconstructions which reflect local moisture supply, and thus local ‘on-the-ground’ conditions, rather than those which may reflect more distal climatic signals (such as marine cores). The selection of proxies reflecting local precipitation or hydrological activity allows the extrapolation of this dated moisture to the catchment the archives are located within (for example, dated fluvial activity reflects hydrological activation within the catchment the river drains). By displaying all broadly contemporaneous humid catchments spatially [49] we therefore evaluate whether potential broad ‘corridors’ of humid conditions may have existed at different points during the Pleistocene. Where dated proxy records reflect overland flow or precipitation from upland areas, catchments adjacent to those containing the proxy archive and containing hydrological networks originating in the same uplands were also displayed as likely humid.

### Climate model data

The paleoenvironmental proxy record for regions such as Iran is typically fragmentary in spatial and temporal coverage, given challenges of sampling bias and preservation. Therefore, to complement the proxy data, and obtain additional insights into the past climate of the region for those periods where proxy records suggest regional climatic amelioration, we also examined precipitation estimates from two climate models. The first was a model produced by Otto-Bliesner et al. [60] for 130 ka, and the second is the recent high temporal resolution (1000 ka time steps) dataset of Krapp et al. [61], covering the last 800 ka. This model is statistically derived from linear regressions based on a HADCM3 climate model for the last 120 ka [62].

## Results

### Paleoenvironmental synthesis

A variety of paleoclimatic proxy records such as fluvial and lacustrine sediments, paleosols, loess and speleothems indicate that diverse and fluctuating paleoclimatic conditions prevailed in Iran over the Quaternary. These are discussed below for northern, central, and southern Iran.

#### Northern Iran

**Fig 3:**
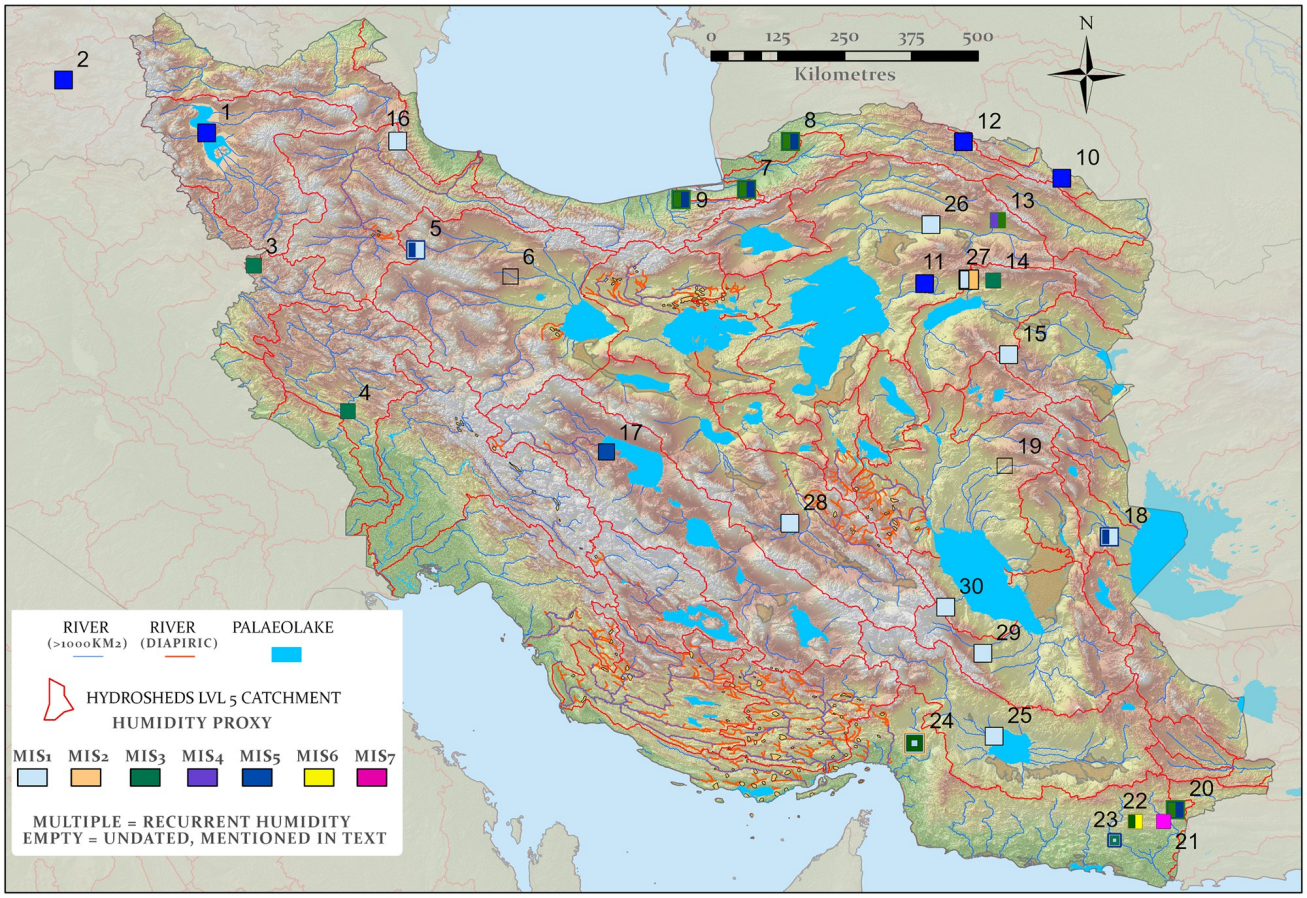
Mapped Iranian paleohydrology superimposed on an elevation map of Iran (Green low, white high). The locations of climatic proxy sites discussed in the text are displayed, numbered and, if dated, color differentiated by the Marine Isotope Stage to which they relate (S2 Table); [1] Lake Urmia, [2] Lake Van, [3] Lake Zeribar, [4] Mirabad lake, [5] Qaleh Kurd cave, [6] Zarand Playa, [7] Toshan, [8] Agh Band, [9] Neka-Abelou, [10] Kalat-e Naderi, [11] Pir Ghar cave, [12] Kopet Dagh Mountain fans, [13] Neyshabour fans, [14] Kashmar fans, [15] Nimbluk, [16] Neor Lake, [17] Isfahan, [18] Sefidabeh, [19] Gavkhoni, [20–23] Makran terraces, [24] Minab fans and terraces, [25] Jazmurian Playa, [26] Sabzevar fan, [27] Kashmar (Shesh Taraz) fans and terraces, [28] Anar fan, [29] Bam fan, [30] South Golbaf basin. Map created by one of our authors (PSB).

In northern Iran, the Urmia Lake pollen record (Fig 3, Site 1; see also Fig 6) suggests major environmental changes between MIS 6 and the Holocene (MIS 1) indicating significant changes in freshwater supply in line with global glacial/interglacial cycles [63]. During interglacials (i.e., MIS 7a, MIS 5e and MIS 5c) with more precipitation, the regions vegetation was characterized by an abundance of arboreal pollen, dominated by *Quercus*, *Juniperus* and *Pistacia* [63]. In contrast, more arid glacials were characterized by semi-arid shrublands (*Artemisia*) and low lake levels [63]. The Urmia pollen record correlates well with that of Lake Van (Fig 3, Site 2), located in nearby eastern Turkey [64], which also shows generally higher lake levels during interglacial phases such as MIS 5, and lower lake levels in glacial phases, including MIS 4 and 2 [65], mirroring wetter and drier hydroclimates in these periods, respectively. During MIS 3, the Van lake level is highly variable, signifying fluctuations in regional hydroclimate [65]. Pollen studies at Zeribar and Mirabad lakes located along the northwestern Zagros Mountains (Fig 3, Sites 3 and 4) show similar ecological fluctuations during the last ⁓40 ka, with an expansion of *Quercus* and *Pistacia* during interglacials and development of more steppic vegetation in glacials [63, 66, 67]. Immediately east of the northern Zagros, lacustrine and palustrine deposits of Zarand Playa (Fig 3, Site 6) vary from fresh to brackish in response to changing moisture availability [68]. Unfortunately, absolute chronologies for these events remain undetermined. Early Holocene lacustrine deposits have also been identified at Nimbluk (Fig 3, Site 15), dating to ~9–8 ka [69], and in the Alborz at Neor Lake where a peat sequence also reflects wet early Holocene conditions contemporaneous with peak insolation at this time [70] (Fig 3, Site 16).

**Fig 4:**
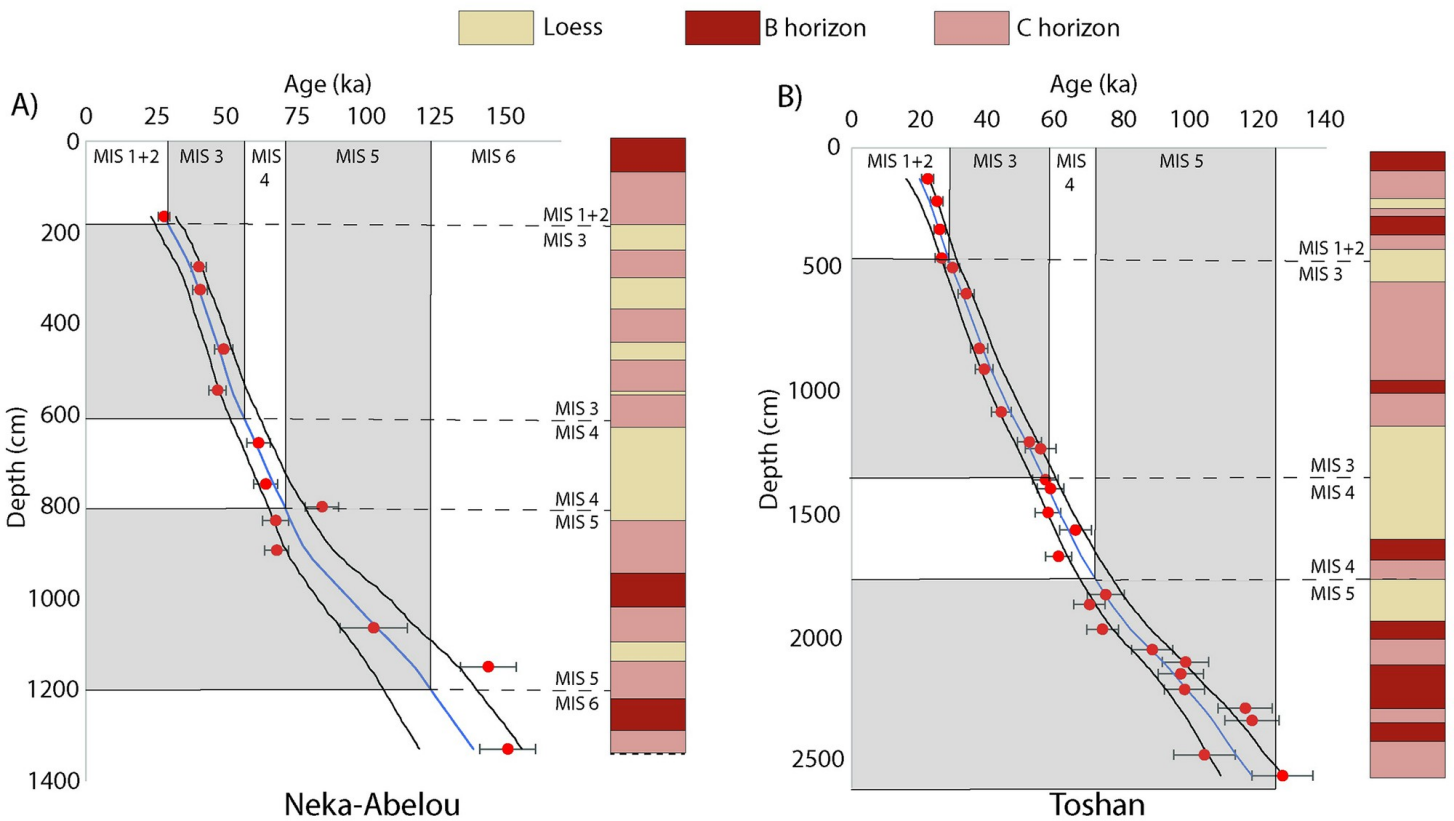
Age-depth modelling results, luminescence ages and simplified loess-paleosol stratigraphy from the Neka-Abelou (A) and Toshan (B) sites, southern Caspian Lowlands, northern Iran. Age-depth modelling was conducted at 1 cm depth resolution using the rBacon Bayesian age model [76] under default priors except for ’thick’, which was set to 30. The blue line represents the modelled median age, while black lines represent minimum and maximum 95% confidence ranges. Luminescence ages from A) Neka-Abelou (fading corrected pIR IRSL at 225 C) and B) Toshan (non-fading corrected pIR IRSL at 290 C) are taken from Kehl et al. [72] and Lauer et al. [73] respectively, and shown as red circles with 1 sigma error uncertainties. Simplified loess and soil stratigraphic logs for the sites are taken from Kehl et al. [72] and Lauer et al. [73]. Marine oxygen isotope stage (MIS) boundaries are shown by age [76, 82], with equivalent depths on the age-depth and stratigraphic plots being inferred from intercept with the median of the age depth model. MIS 3 and 5 are shaded grey.

Environmental fluctuations from the Middle Pleistocene to Holocene have also been reconstructed from loess profiles in northern Iran, which generally agree that drier and colder periods were characterized by heightened aeolian activity and dust accumulation, while relatively stable conditions prevailed during warmer, wetter periods [71–73]. Along the northern foothills of the Alborz Mountains and bordering the deserts of Turkmenistan, multiple loess sequences have been reported, including Toshan, Agh Band, and Neka-Abelou (Fig 3, Sites 7–9). These indicate a highly dynamic climate from the late Middle Pleistocene (since ⁓200 ka) to the Holocene [72, 74, 75]. Robust chronologies have long been wanting from these sites, but recent work based on luminescence dating allowed relatively detailed inferences regarding the timing of shifts in hydroclimate [72]. A Loess-soil stratigraphy and Bayesian age-depth models calculated by the authors using rBacon [76] for the Toshan and Neka-Abelou sequences (Fig 4), together with the published studies for these sites, illustrates the likely timescales of wetter conditions as documented by paleosol formation. Relatively well-developed paleosols formed in MIS 5a, 5c and especially 5e, with forest or steppe conditions and warmer, wetter climates, potentially driven by precessional insolation peaks, and MIS 5e likely warmer and wetter compared to today. Soil carbonate stable isotope and mineral magnetic analyses of the MIS 5e soil suggest particularly wet winters and hot dry summers [77], perhaps evidence of enhanced winter moisture input via the Westerlies. MIS 5c and 5a also show evidence for strong soil formation, indicating enhanced westerly winter moisture, with comparisons to Holocene soils suggesting similar conditions [72]. The climate during MIS 4 was considerably drier, and enhanced dust accumulation lead to the formation of thick loess deposits that remained relatively unweathered. These climatic trends correspond with those observed in Lake Urmia [73], suggesting relatively coherent climatic dynamics across northern Iran during the Late Pleistocene. Perhaps the most striking feature in these loess records is the numerous phases of soil development occurring at multiple sites during MIS 3 and partly MIS 2, at the same time as relatively high loess accumulation rates [77]. The Neka-Abelou sequence shows these multiple soil formation episodes during MIS 3 (Fig 4a), though soil development is weaker than during MIS 5 and 1, the latter of which overprints MIS 2 loess. These fluctuations are also seen in multiple climate proxies derived from the loess sequences and appear to reflect regional pluvial events interrupting otherwise arid conditions in the area [72]. As such, MIS 3 appears to be have been characterized by considerable centennial-millennial scale climatic instability in the Caspian Lowland, with evidence for multiple phases of wetter conditions as also suggested by the Lake Van record [65]. Chronological uncertainties and differential preservation of soils at different loess profiles prevents specific wider correlations for these soils, but the fluctuations may be linked to millennial-scale hemispheric Dansgaard-Oeschger climate fluctuations, seen particularly in the Greenland ice cores, although they may also be influenced by changing water levels in the nearby Caspian Sea [74, 75]. Overall though, despite these pluvial episodes during MIS 3, the loess records show a tendency to extreme drought during the last glacial period, particularly during MIS 4 and 2 [77]. Further east, close to the border with Turkmenistan and Afghanistan, loess-like deposits are also found covering a similar time interval and intercalated with fluvial sediments [71]. Unfortunately, the chronologies of these sequences (Fig 3 Site 10) are far less secure, though paleosols from at least MIS 5 are preserved, hinting at moister interglacial conditions in this otherwise arid region. This pattern is more pronounced further west along the Alborz foothills [71].

**Fig 5:**
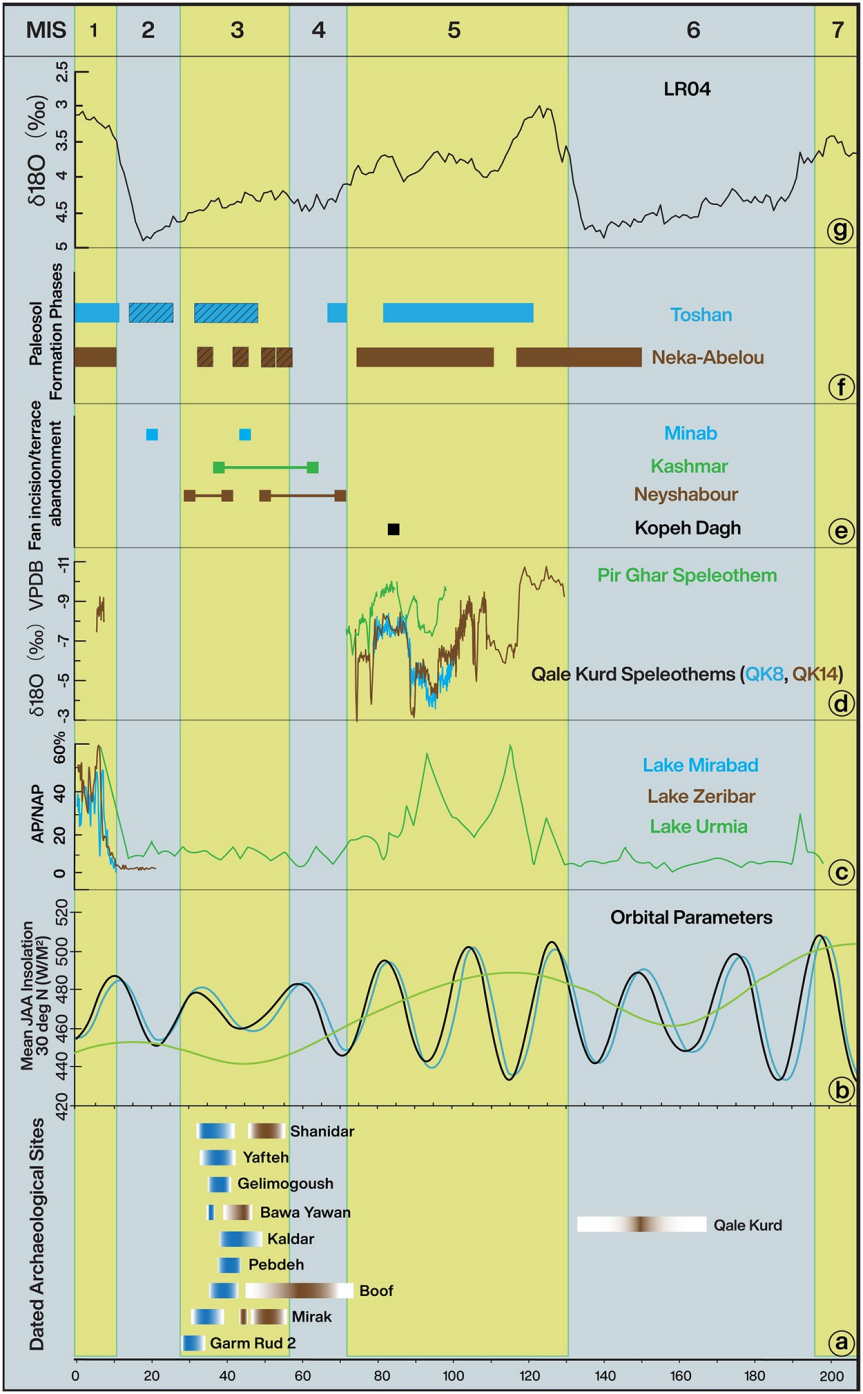
The relationship between dated archaeological sites in Iran and diverse regional environmental proxies. A) Dated archaeological sites with Middle (brown) and Upper (blue) Paleolithic affinities. B) Orbital parameters (eccentricity, green; and precession, blue) which influence local insolation receipt (black). C) Pollen record from Lake Urmia, Zeribar and Mirabad lakes from along the northern to central Zagros, showing arboreal to non-arboreal pollen ratio, suggestive of forest/shrubs vegetation during MIS 5 and 1 [63, 67, 93]. D) Speleothem studies from Qaleh Kurd in western and Pir Ghar in the eastern Iranian Plateau, showing wetter conditions during phases of MIS 5 and also the Holocene [44, 78]. E) Alluvial fan and river terrace abandonment [80, 81]. F) Paleosol formation phases, weakly developed loess soils are shown by black diagonal lines [72, 73]. G) Oxygen isotope values from the LR04 global benthic stack [82].

Speleothem records provide detailed insights into terrestrial paleoenvironmental conditions in northern Iran during periods with a positive moisture balance. The Qaleh Kurd cave (with a record covering 127–73 ka and 7.5–6.5 ka) in NW Iran (Fig 3 Site 5), and Pir Ghar cave (with a stalagmite record spanning 100–70 ka) in the NE of the country (Fig 3 Site 11), provide key information, especially for MIS 5 [44, 78]. Both reconstructions indicate multiple wet phases during MIS 5 (Fig 5), comparable to the Lake Urmia profile and the loess records. Comparison of both records confirms strong similarities, with both showing fluctuations resembling those observed in the marine isotope record (Fig 5g) as well as with the Soreq and Sanbao cave δ^18^O profiles from western and eastern Asia [44], suggesting a common atmospheric teleconnection. It thus seems plausible that the position of the westerlies, and the pan-regional circulation dynamics that link the Mediterranean with the Asian realm, governed the moisture budget and precipitation seasonality across the northern Iranian Plateau. The lack of speleothem growth between MIS 5 and 1 suggests rainfall was below 300 mm. This is enough to form the weak soils that develop in loess during this period, but puts an upper limit on the rainfall northern Iran received at this time.

Wetter conditions during MIS 5 in northern Iran are further suggested by alluvial fan abandonment ages, which have been interpreted as providing maximum ages for humid periods. During the Holocene abandonment is thought to occur upon the onset of local pluvial conditions and is followed by fan incision, dissection and river terracing [79], and this has tentatively been extrapolated further into the Pleistocene [79]. Abandonment occurs in the Kopet Dagh Mountains (Fig 3, Site 12) at ~83 ka [80]. Two generations of abandonment also occurred at Neyshabour between ~70–50 ka and 40–30 ka, and at Kashmar between 62–38 ka and 61–41 ka [81] (Fig 3, Sites 12–14), potentially suggesting abandonments in both MIS 5 and 3, though the errors are large and MIS 4 is also a possibility.

#### Central Iran

In central Iran, paleosols provide some of the oldest records of hydroclimatic changes. Much wetter soil formation phases are found in eastern Isfahan during the Early Pleistocene to the late Middle Pleistocene, tentatively attributed to >412 ka and between MIS 11 and MIS 5c (Fig 3, Site 17) [83]. Their carbonate versus clay ratio and δ^13^C values suggest >500 mm of annual rainfall during these wet phases, with the earlier soils being wetter. The limited vertebrate records from the Zagros and northern Dasht-I-Kavir regions also support a markedly wetter Mid-Late Pleistocene climate [84]. A range of large mammals, including Equids and Rhinoceros, have been found here which indicate enhanced biomass and wetter environments during a Mid-Late Pleistocene interglacial, although the chronology is based on a relative taxonomic date only.

Lake deposits at Sefidabeh in the geographically extensive Sistan Basin (Fig 3 Site 18), dated to MIS 5, formed under substantially wetter conditions than today, though a specific moisture source (monsoons vs westerly) could not be confirmed [85]. Recent geomorphological studies in Afghanistan further confirm a highly dynamic Pleistocene hydrological history for the Sistan/Helmand Basin, with numerous terraces and lake shorelines thought to reflect every glacial/interglacial transition over the last 800 ka, with a broad pattern of increased drying over time [86]. Lake sediments indicate a reactivation of the Sistan lakes during the Holocene, hypothesized to reflect an early Holocene high stand fuelled by both monsoonal and westerly moisture [87], succeeded by a mid-Holocene drying following a southward retreat of the Intertropical convergence zone (ITCZ), and late Holocene low stands after the establishment of modern westerly-dominated pattern [87]. Though this Holocene wetting is concurrent with monsoonal incursions in regions further south (see below), this data does not necessarily represent a major movement of the monsoon northwards across most of Iran. Kutzbach et al. [88] illustrate that enhancement of winter storm tracks and westerly moisture may also occur at precessional peaks, amplifying westerly moisture in regions such as northern Iran, and perhaps increasing it in step with increases in summer moisture in southern Indian Ocean Sothern Monsoon (IOSM) influenced regions.

Evidence for higher moisture availability in nearby areas is provided by undated paleolake high stands at Gavkhoni in the central Zagros, where gypsiferous marls are found atop fan surfaces (Fig 3, Site 19), 150 m above the current playa [56]. Jones et al. [56] speculated that these may represent an Early-Mid Holocene high stand synchronous with the African Humid Period and wetter conditions in Arabia, thus reflecting a northward penetration of the summer monsoon into Iran to ~33°N. However, the contrast between the level of moisture receipt such a high stand would imply, the muted moisture increase seen at this time in the Urmia, Mirabad and Zeribar records [56] and the comparatively limited moisture influx seen at more southerly (~27° N) latitudes in Arabia [89, 90] casts doubt on this interpretation. It seems more likely that this Central Zagros high stand reflects a substantially more humid interglacial period earlier in the Pleistocene, when summer insolation during a precessional peak was greater than the Holocene (Fig 5b). This would allow for more northward ITCZ migration, perhaps with concurrent westerly amplification [88], such as during MIS 5, as indicated by the aforementioned paleolake loess, and speleothem deposits.

#### Southern Iran

Evidence for enhanced Pleistocene water availability in southern Iran is sparse, but compelling. Near the Gulf of Oman, at Makran, a series of terrace abandonment events have been shown to coincide with interglacial and interstadial periods during MIS 1, 3, 5, 6 and 7 [79] (Fig 3, Sites 20–23). Given the southern position of the sampled sites, and the conspicuous timing of these events in relation to monsoonal incursions immediately south and east in Arabia, this has been considered to reflect monsoonal moisture influx into southern Iran. Indeed, some areas of the Makran coast and the southern areas of Pakistan receive monsoon winds and low levels of summer precipitation today, though they lie outside the principal limits of current monsoon precipitation [46, 47]. At Minab, proximal to the Strait of Hormuz (Fig 3, Site 24) fans and terrace surfaces were abandoned at ~20, 44 ka [81] and at the end of the Holocene wet phase at 5.6 ka [81]. Fan abandonment, terracing and lake sediment deposition across much of eastern Iran (Fig 3, Sites 13, 24, 26–30) further suggests widespread wetter conditions, particularly 10 to ~8 ka, although this could relate to enhanced westerly moisture supply, rather than being linked to mid-Holocene monsoonal moisture covering all of Iran.

Additional evidence for Holocene moisture flux comes from paleolakes. A Holocene penetration of the IOSM into southern Iran may be indicated by records from Jazmurian Playa [91]. The sediment profile records variations in fluvial/aeolian inputs and evaporite/illite deposition (Fig 3, Site 25), the timing of which is consistent with increased moisture supply from both monsoonal and westerly sources at different times. During periods of stronger ISM at ~14–13 ka and ~11.4–9.4 ka the playa records wetter conditions, with a lagged drying in the Holocene following monsoon weakening, suggested to relate to continued influx of westerly moisture, and later, a southwards shift of the westerlies [91].

Given the more muted Holocene wet phase compared to earlier wet phases seen immediately to the south in Arabia [92], these observations may be of relevance when we consider Pleistocene insolation peaks. In concert, the Makran, Jazmurian and Hormuz data suggest that the IOSM reached southern Iran under the muted orbital forcing of the Holocene, while a significantly enhanced IOSM may be expected with comparatively greater insolation peaks during some periods in the earlier Pleistocene. Terrace abandonments during MIS 7, 6, and 5 in Makran seem to corroborate this interpretation [79]. It would appear then that repeated monsoonal incursions reached at least the very southeastern portion of Iran repeatedly during the Mid and Late Pleistocene.

Summarizing the Iranian paleoclimate proxy record it is evident that the data is sparse and the spatial distribution is uneven, with fewer proxy records as you move from north to south. Notwithstanding, this in all regions there is evidence for enhanced moisture in MIS 5 and during the Holocene, with some evidence for more moisture during MIS 3, particularly in northern Iran.

### Paleohydrology and Pleistocene water availability

**Fig 6:**
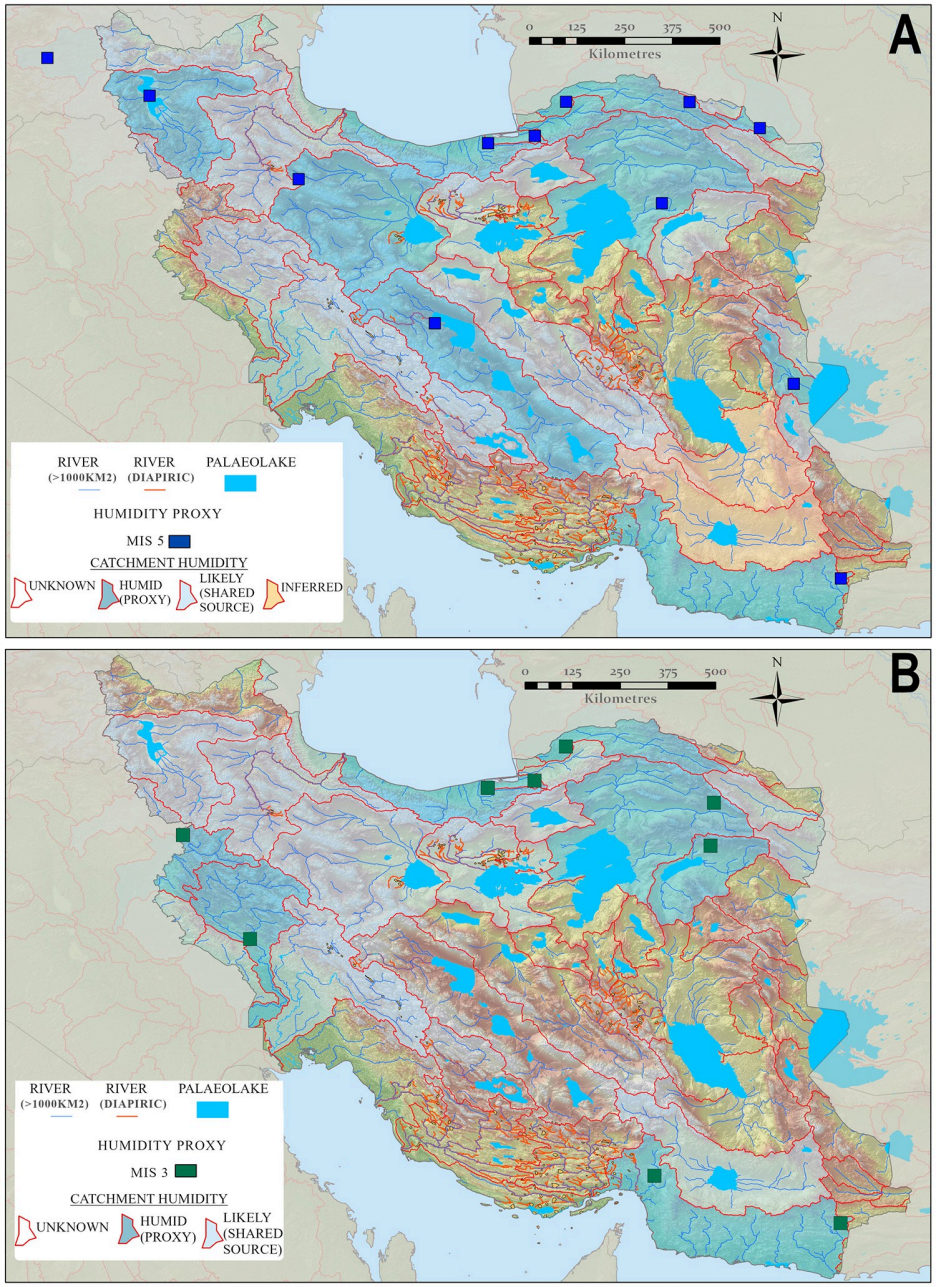
Hydrologically active catchments during MIS 5 (A) and MIS 3 (B), based upon dated proxies which reflect enhanced humidity. Dated proxies such as lakes and rivers reflect an accumulation of overland flow from upland areas, where this is the case adjacent catchments containing hydrological networks originating in the same uplands are also displayed as likely humid. Map created by one of our authors (PSB).

Our paleohydrological analyses identified 145,354 km of rivers and 115 paleolakes calculated from 6380 paleolake deposits. Only a handful of these paleolakes have so far been studied (Fig 3). The distribution of these mapped water bodies in relation to the archaeological data (Fig 6) suggests that most archaeological sites are found close to rivers and lakes. The country’s topography means that the principal concentration of large lakes occurs in the numerous endorheic basins on the Iranian Plateau. Several particularly large paleolakes are found across the northern Dasht-I Kavir, fed by drainage systems originating from deeper in the arid interior and also from the Zagros, Alborz and Kopet Dagh Mountains. South of these, several smaller paleolakes are identified resolutely within the current arid core of the country, while in the east, a large paleolake is identified in the Sistan Basin. In the west and south, a series of large spinal basins running northwest to southeast along the Zagros contain numerous paleolakes, and a few are lakes are found in the south such as Jazmurian playa that lies to the south of the Lut, in the southeast of Iran (Fig 3, site 25).

Our analyses support the Lut Desert in south-central Iran being a huge endorheic basin that once contained a large paleolake. The region is notable for the presence of some of the largest yardangs (streamlined elongated ridges of sediment sculpted by wind erosion) in the world, standing up to 70 m from the basin floor and distributed over an area of ~6870 km^2^ [94]. Several prior studies, using aerial and sedimentological methods indicated that these yardangs represent the eroded remnants of a former paleolake of Pliocene or Early Pleistocene age, with subsequent phases of aggradation, base level change and erosion leading to their current morphology [95, 96]. While the geochronology of the yardangs and their paleoclimatic record is yet to be determined, their detection by our method further corroborates a lacustrine origin. We calculate that Lut Lake had an area of 16,226 km^2^, closely matching earlier findings [96, 97]. It is important to note that Krinsley et al. [95] suggest that the internal stratigraphy of the yardangs indicates a saline character for this lake. Our analyses of the geology and paleohydrology of the catchments suggest that up to a third of its radial drainage crosses diapiric terrains. The large exposures of highly soluble salts and the numerous saline springs found in these regions could explain its saline nature. Whether this lake dated to the Pliocene or Pleistocene remains unclear. However even today, a series of smaller saline playas occur on the basin floor between the yardang ridges, which suggests that after the period of erosion of the former lake sediments a smaller complex of lakes occupied the basin floor when enhanced precipitation reached the region [97].

### Linking hydrological and proxy data

While the diachronic paleohydrological mapping highlights numerous water sources, in the absence of a substantial field dating program, inferences of when these were individually active depends upon the hydrological proxy records outlined above, or on paleoclimate modelling. Available proxy data discussed above is spatially and temporally sparse. Nonetheless, the records can be linked to the paleohydrological maps via the catchments in which they are located (Fig 3). These data illustrate that the moisture supply evidenced by the lake, fluvial, speleothem and loess records discussed above appears to provide a coherent network of drainages exiting the Zagros, Alborz and Kopet Dagh Mountains during multiple periods in MIS 5 and MIS 3, ending in the terminal paleolakes on the Iranian Plateau (Fig 6). As a result, a chain of playas running east-west across the northern Dasht-I Kavir could have been extensive lakes during the MIS 5, which in concert with their southward-draining feeder rivers that exited the Alborz and Kopet Dagh, provided a connected corridor of relatively close water sources. The same playas and drainage systems likely also held moisture periodically during MIS 3, although probably to a somewhat lesser extent. It should also be noted that the conservative threshold we used for drainage (>1000 km^2^ upstream area) hides many of the initiating tributaries in the mountains where this moisture is documented, and hence may underestimate true connectivity. Notwithstanding this the results support routes B and C of Vahdati Nasab et al. [13] as viable options for hominin dispersal at that time (Fig 1) These two distinct routes were part of a broad region of ameliorated environments in northern Iran that from now on we will call the ‘northern route’. These results suggest a recurring opening of this northern route that connects the Zagros, Alborz, Kopet Dagh, Caspian and Dasht-I Kavir regions to Central Asia to the east and the Caucasus, Taurus Mountains and Levant to the west.

The Zagros shows evidence for relatively higher moisture supply compared to the plateau during all periods, highlighting their potential role as a refugium throughout much of the Pleistocene and episodically connected to the regions to the east and west by the aforementioned northern route. Interestingly moisture availability during MIS 5 may have opened another hydrological corridor, stretching south-eastwards from the Zagros to the Makran and Baluchestan regions towards Southern Asia (Fig 6). This route is suggested by fluvial activity at the Makran terrace sites [98, 99] and the Holocene record of Jazmurian Playa. As the monsoon reached the latitude Jazmurian Playa during the relatively weak mid-Holocene wet phase, it is reasonable to assume it would have done so during the more intense humidity of MIS 5, allowing the Lut region to be traversed from the Zagros to the Sistan lakes. We term this newly recognized route the ‘southern inland route’ in order to distinguish it from the southern coastal route that has previously been suggested by numerous authors [100–102].

### Climate modelling

Based on hydrological mapping and on-the-ground proxies, we identify two broad periods (MIS 3 and 5) when large portions of Iran could have been traversed without dangerously leaving freshwater sources behind. However, given the paucity of dated paleoclimate proxy data in this region we evaluate to what extend paleoclimate models can inform us about the rainfall regimes when the paleoclimate records suggest wetter conditions in Iran.

**Fig 7:**
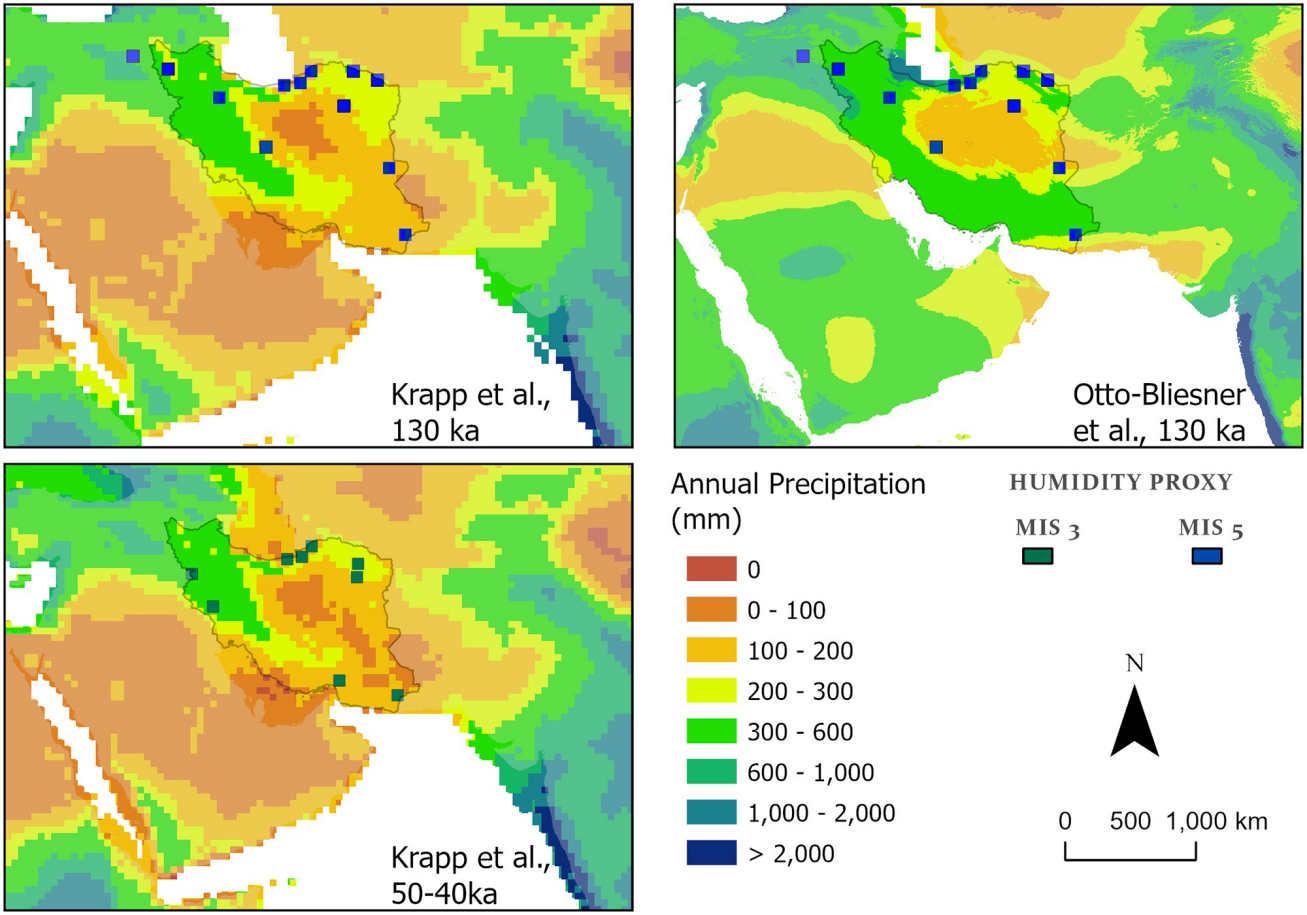
Annual precipitation estimates from paleoclimate models for MIS 5e (~130 ka Krapp et al. [61], and ~130 ka Otto-Bliesner et al. [60]), and MIS 3, overlain with broadly contemporaneous proxy sites. MIS 3 data is from the Krapp et al. [61] model and displays the mean value during the broad window of 50–40 ka when age depth modelling of northern Iranian loess (Fig 4) suggests phases of enhanced humidity. Map created by one of our authors (PSB).

For MIS 5e, climate models show strikingly varying results (Fig 7). In broad terms the Krapp et al. [61] model could be described as ‘dry’, illustrating most of Iran except the Zagros, Alborz and Kopet Dagh Mountains to be desert (< 200 mm of annual rainfall). In contrast, the Otto-Bliesner model [60] suggests substantial precipitation across much of the country, with only a relatively small arid central region around the northern Lut/southern Dasht-I Kavir deserts and improved moisture supply to the north and east suggesting strong westerly rainfall, as well as southern Iran, likely associated with monsoonal rainfall. The Krapp et al. model is not supported by the proxy data whilst that of Otto-Bliesner [60] is. This latter model is in agreement with proxy evidence with the speleothem and loess records demonstrating the presence of strongly enhanced rainfall in northern Iran. The increased MIS 5 moisture supply indicated by fan and terrace abandonment dates in the north-eastern and southern regions, and by lake formation in the Sistan Basin is also mirrored by this model, as is the wetter MIS 5 in the Zagros Mountains suggested by the Lake Urmia pollen data. This depiction of precipitation patterns thus appears to closely match the distribution of MIS 5 proxy records. It also suggests a band of moisture in southern Iran that during MIS 5 reached as far northeast as the Jazmurian playa, as we tentatively suggest in the previous section. It also captures the minimum latitudinal extent of MIS 5 monsoonal moisture indicated by field investigations in adjacent regions including Arabia [35, 103].

Consequently, we suggest that the Otto-Bliesner model may more accurately reflect conditions during MIS 5. Interestingly, the former model reinforces the broad picture from the proxy records that despite the potential traversability of southern inland and northern routes (at least during MIS 5e) the core of the Iranian Plateau deserts likely remained arid. This argues for a coherent separation of moisture sources producing the two routes, as suggested by the speleothem data and their latitudinal positions. The northern route is dominated by westerly rainfall and a southern inland route by monsoonal one. This suggest that two routes were open and more accessible during different seasons. The northern route would have been a winter/spring (westerly) route, whereas the southern route would have been open in the summer when the IOSM delivered rainfall.

For the MIS 3 the only available climate model was that of Krapp et al. [61]. This ‘drier’ model implies the Zagros to be habitable between 70 and 30 ka, and captures the enhanced moisture across northern Iran during 50–40 ka suggesting that the northern route was open at this time. This coincides with the broad period when the age-depth model from loess sections (Fig 4) indicates phases of incipient soil formation. Although these pluvial phases recorded in MIS 3 loess soils would not have been as strong or long-lived as during MIS 5, they do suggest the potential periodic recurrence of a brief ‘northern route’ and this is to some extent also supported by fan incision data of Thomas and Fattahi [81]. This suggests that the northern route provided periodic connectivity between the Zagros Mountains and regions to the east in MIS 3 (Fig 6). However, although enhanced moisture availability in northern Arabia associated with the ~55 ka insolation peak has been interpreted to reflect a northward shift of the summer monsoon at this time [35, 104], and a limited penetration of southern Iran by this moisture may be supported by proxy records, a southern inland route from the Zagros Mountains to the east cannot be demonstrated during MIS 3 at present, either by the catchment analyses or climate models.

## Comparison of archaeological records and paleohydrology

### Distance from water sources

**Fig 8:**
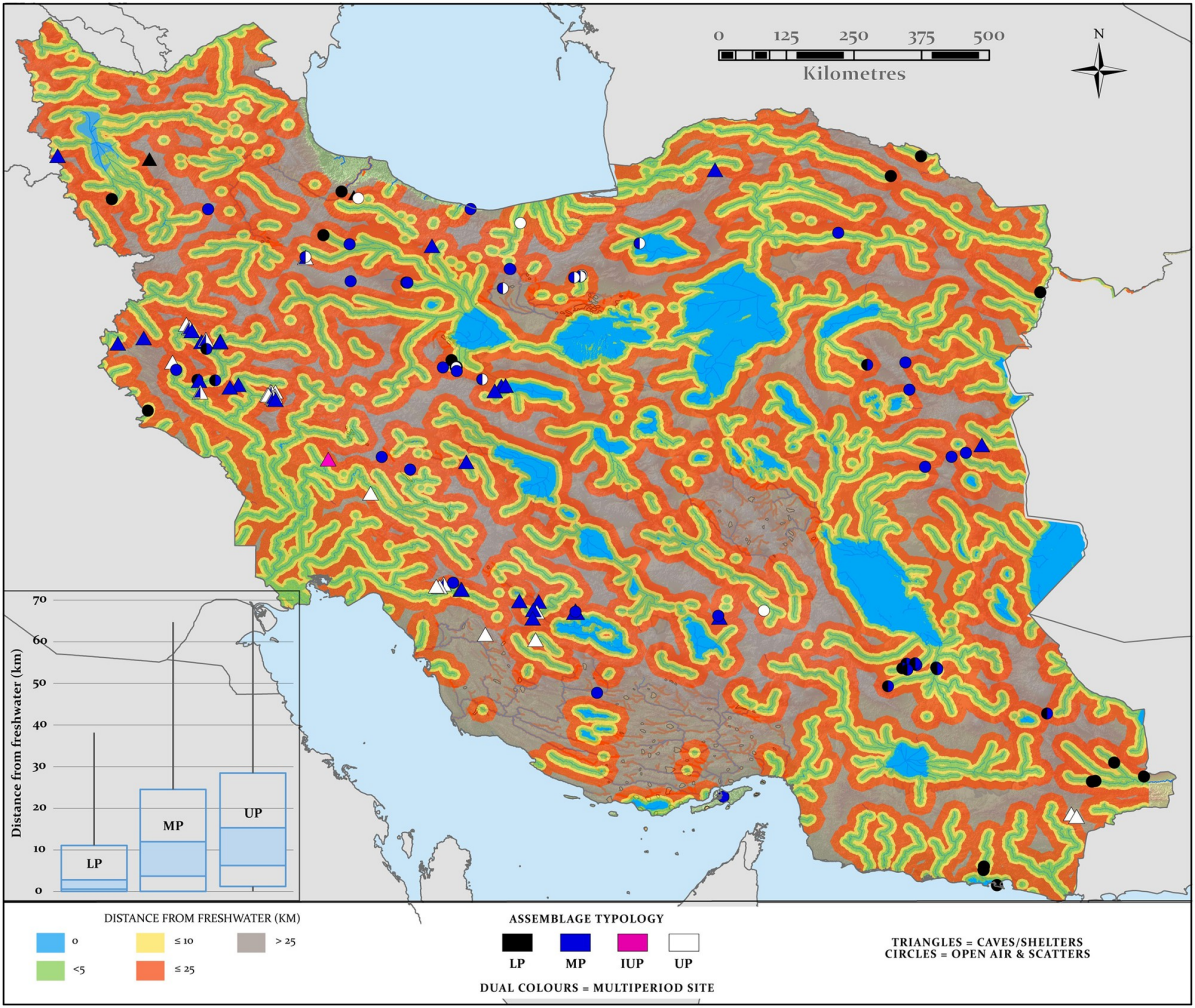
Distance from water analyses highlighting the distance from mapped paleohydrology for all areas of the country. The inset graph shows box plots of the distance from water of LP, MP and UP sites. Map created by one of our authors (PSB).

To evaluate the role that paleohydrology played in promoting hominin occupation and dispersal we evaluated the affinity of the LP, MP and UP site’s to water by performing a distance-from-water analysis. In the absence of any detailed sedimentological data from the mapped lakes to assess their changing conditions, such analyses assume water potability. However, the condition of such water bodies would be variable in response to presence of salt rich rocks within their catchments, levels of precipitation, evapotranspiration, the size, morphometry and hypsometry of their catchments and drainages [50]. Several Iranian rivers today exhibit high salinity due to extensive surface exposures of salt diapirs and salt glaciers [97, 105]. This would have influenced the salinity of water bodies downstream, sometimes rendering them undrinkable. While the chronology of their exposure remains to be clarified, it seems likely that salt bodies have been present at the surface through much of the Pleistocene and thus have been influencing the salinity of the catchments they are in for some time [97]. Therefore, we have re-classified as saline any water course which occurs within 5 km of a salt body documented in the USGS geological map of Iran [106] (Figs 3, 6, and 8). The primary regions where these rivers occur is in the southwestern Zagros and northwestern Dasht-I Kavir. This is relevant for the potential of these regions as routes for hominin movements (Fig 1), as the question becomes whether sufficient atmospheric moisture could dilute the concentration of salts to a potable level, or alternatively if these would be permanently unpotable waters even during periods when the rivers were perennial. In the interest of remaining conservative when calculating distance between water sources we have assumed the latter and excluded such rivers from the distance analysis.

Distance calculations were used to determine the distance from modelled freshwater sources to the archaeological sites in our database. Areas that exceed 25 km from water may have been more problematic to traverse, as few sites of any period occur beyond this distance. LP sites (N = 35) have the lowest average distance to water (less than 2 km) when compared to sites of other periods (Figs 8 and 10), in keeping with patterns observed in Arabia [48, 49], and high levels of water tethering observed for Acheulean hominins in Africa [107]. However, water is not the only important factor determining the location of LP sites as they are also situated close to raw material sources, such as chert and flint outcrops in the Zagros Mountains and on river terraces of eastern Iran where quartz, quartzite and tuff are abundant [18]. These early sites appear mostly at lower elevations where lakes and permanent rivers would also have been abundant, comparable to LP sites in the similar settings of the Arabian Peninsula [108] and Levant [109]. In contrast, MP sites (N = 82) appear slightly less tightly tethered to open water bodies and occupy some zones currently devoid of LP sites such as the high Zagros Mountains. Both LP and MP sites occur deeper within the current desert regions than later sites, with the tentative suggestion of MP sites reaching further into the desert core than LP sites. This pattern may reflect greater water availability in these deserts during humid phases of the Early and Middle Pleistocene than those of the Late Pleistocene, facilitating occupations. UP sites (N = 41) in contrast exhibit the greatest range and largest average distance from water (Fig 8). These patterns likely reflect a range of influences upon site location, of which freshwater, while critical, was only one. While survey bias cannot be discounted, they likely also reflect occupation and foraging strategies fluctuating through the Quaternary in accordance with the environmental tolerances and preferences of different hominins, shifting ecological conditions and range variation responses over timescales varying from seasonal to millennial (for example in accordance with the distributions of prey), and technological innovations enabling hominins in the MP and UP to more intensely exploit the environment and expand their hunting and foraging ranges.

The national-scale modelling of distance from water also presents the potential to evaluate routes for dispersal, if distance bands are considered spatially as networks through which routes at minimal distances from water are sought. This is of course diachronic data, and the results of the climate proxy and modelling data should be considered in terms of placing timestamps on the likely periods when any parts of these networks were active. *H*. *sapiens* requires abundant water to offset losses, particularly when foraging in hot climates, and there is little data to suggest other hominins were less water dependent. Though by no means a perfect analogue for earlier *H*. *sapiens* populations or earlier hominins, as an indicator, recent forager groups often remain in close proximity to water for daily access and have high seasonal range variations in accordance with the changing distribution of water [110]. This tethering effect, while obvious as water is key to survival, is also supported by the data presented here as most sites for each period are located less than 15 km from water (Fig 8).

When determining possible routes using distance to water it is worth considering that although some gaps are present with inter-drainage distances in excess of 25 km, the chosen upstream area threshold of 1000 km^2^ functionally excludes river headwaters and minor tributaries. Thus, the chosen conservative threshold captures only larger systems more likely to have been incised and repeatedly reactivated along the same course. This may artificially create some larger gaps between catchments that may not have existed. Notwithstanding these limitations the distance from water analysis suggest similar northern and southern routes as defined by the catchment and paleoclimate model analysis outlined above. From the Lake Urmia region (Fig 8) rivers connect to the Zagros spinal chain of lakes and highland drainages and this facilitates movements south along the high mountains and onwards into southern Iran. The Lake Urmia region also links to two routes across northern Iran. One is from the Zagros to the Caspian Sea and then onwards to the northern Kopet Dagh Mountains whiles the other connects to the rivers flowing southwards from the Alborz into the lakes in the northern Dasht-I Kavir and onwards along the southern Kopet Dagh.

**Fig 9:**
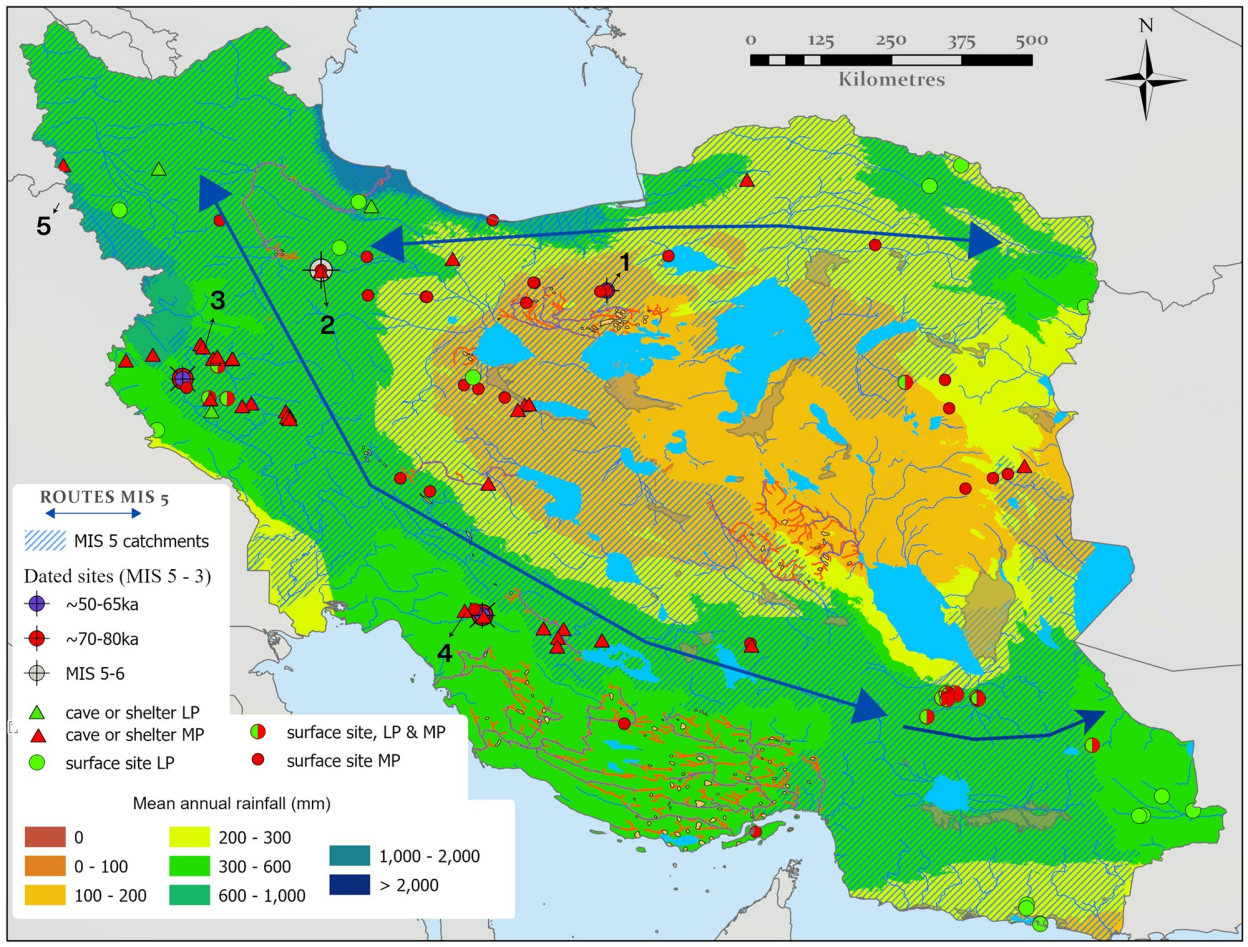
The distribution of LP and MP archaeological sites in relation to MIS 5 paleoclimate data. Sites which are discussed are numbered as follows; 1.Mirak, 2.Qaleh Kurd, 3.Bawa Yawan, 4.Ghar-e Boof, 5.Shanidar. The base map is the Otto-Bliesner et al. (130 ka) mean precipitation climate model [60]. This is overlain by catchments where MIS 5 humidity and active hydrology (hatched) are suggested by our data (see Fig 6). Simple representation of the broad routes through Iran are overlain as arrows. Map created by one of our authors (PSB).

These results complement the proxy and model data, adding nuance to both the hypothesized northern and southern inland routes. This is particularly true for the northern route, as they suggest a potentially significant role for the network of northern playas and their drainages in facilitating east-west dispersals, despite being located on the desert fringe in model outputs (Fig 9) and distal from the highland loess proxies. Any potential central Iran route through the Dasht-I Kavir and Lut Deserts is however not supported by the distance to water data or by the proxy or modelling data outlined above.

### Archaeological evidence for dispersal routes

**Fig 10:**
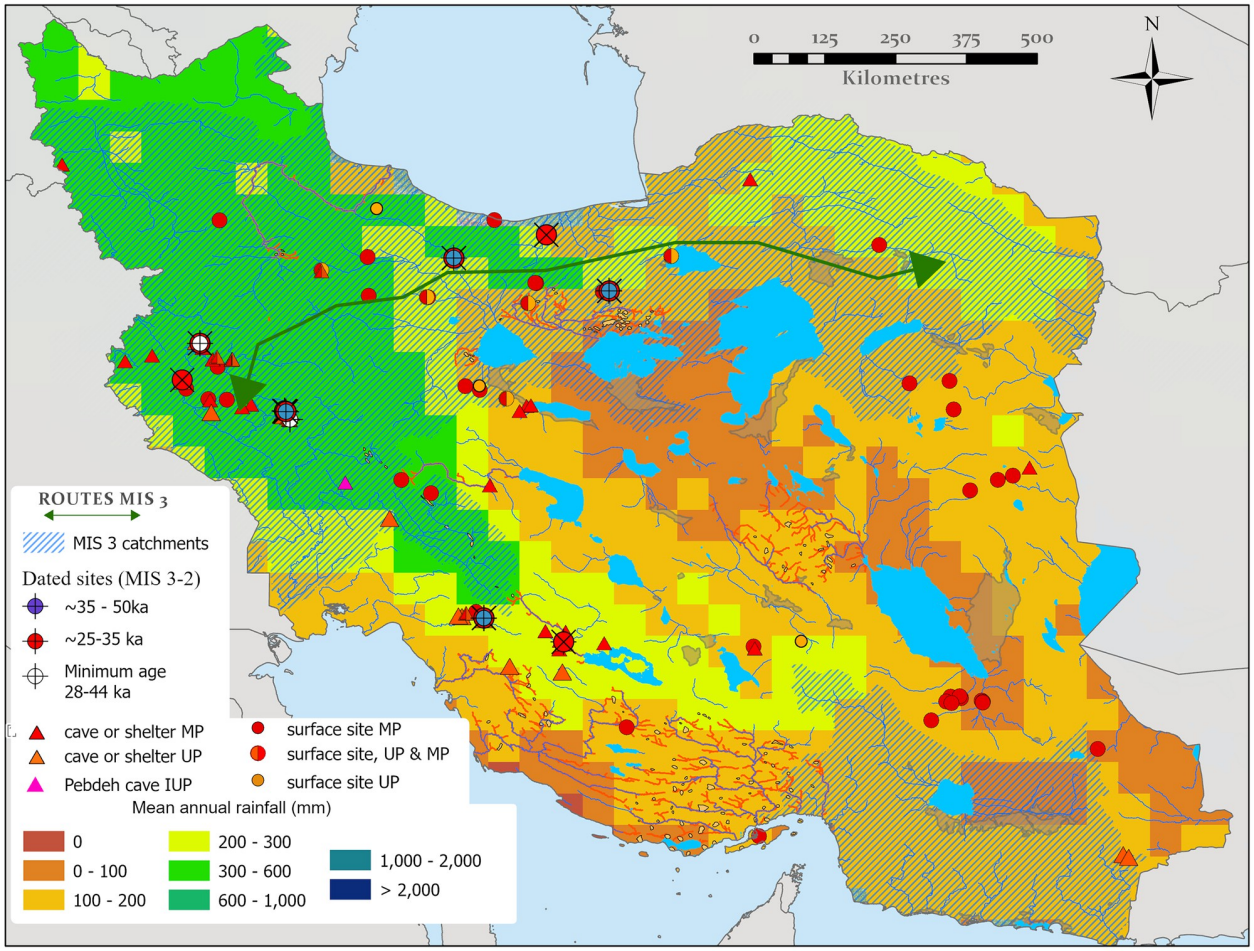
The distribution of MP and UP sites in relation to MIS 3 data. The base map is the Krapp et al. (50–40 ka) mean precipitation climate model [61], and is overlain by catchments where MIS 3 humidity and active hydrology (hatched) are suggested by our data (see Fig 6). Map created by one of our authors (PSB).

Combining archaeological records with our paleohydrological synthesis can help to identify evidence for the exploitation of the dispersal routes identified above. However, there are caveats. The number of Paleolithic sites in Iran is small, they are unequally distributed and influenced by sampling bias. Systematic archaeological field surveys focusing on the Paleolithic have mostly been conducted in regions along the Zagros Mountains as this region has gained particular prominence in discussions of Iranian archaeology, hosting a few known hominin fossil collections in the region [7, 25, 27, 111]. Relatively few surveys have been initiated along the Alborz Mountains, the Iranian Central Desert, and the northern shores of the Persian Gulf. For the LP, the scarcity of sites and paucity of paleoenvironmental proxy records precludes a direct comparison to the paleohydrology. Yet for the MP and UP, proxy records, paleoclimate models and dated archaeological sites exist and evaluations can be made. This has been achieved by overlaying the archaeology onto the maps of paleohydrology and highlighting catchments that either the proxy records or the paleohydrological models suggest are humid, thus allowing a comparison between humid regions and hominin occupation (Figs 9 and 10).

Although a considerable number of MP sites have been identified in Iran, only four are from dated contexts, ranging from ⁓150–42 ka. As a consequence, early MP dates are absent and the onset of MP is poorly understood. Notwithstanding this, MP dispersal is likely as it is found in the Levant and Arabia during MIS 5, climatically ameliorated dispersal routes have been identified at this time, and MP sites appear to be preferentially located along these routes (Fig 9). As a whole, most MP sites are in regions where there is evidence for substantial water availability in MIS 5, rather than MIS 3 (Fig 10). These MP sites are found in diverse settings across Iran, including caves, rock shelters, and as open-air sites [18]. While substantial work has focused upon caves and shelters, the importance of open-air sites has recently been highlighted by the discoveries of Chah-e Jam, Soufi Abad, Moghanak, Otchounak, and Qaleh Gusheh [12, 14–17], which except for Moghanak and Otchounak, are all located near water resources.

The Qaleh Kurd cave MP assemblage is dated to ⁓150 ka [26], suggesting the operation of northern routes during MIS 5, but the scarcity of sites dating to this period is a significant gap in our understanding of dispersals. As depicted in Fig 10, MP sites are present in the western and southern Zagros Mountains and in the southern Lut desert. Undated open-air sites have yielded MP flake-based lithic technology including common usage of Levallois techniques with availability of handaxes [112, 113]. MP sites are located in the vicinity of paleolakes or ancient water sources [114–116], which were probably related to MIS 5 moisture availability, though without dates, this can not be verified as of yet.

Dated MP sites from within the Zagros such as, Ghar-e Boof, Shanidar and Bawa Yawan range to ⁓77–42 ka [7, 25, 117], including fossils of Neanderthals, reflecting a habitable Zagros during most of MIS 4 and 3. The dated MP sites provide support for a MIS 3 dispersal; for example, the open-air site of Mirak, situated on the northern edge of the Iranian Central Desert. Mirak has produced a stratified MP assemblage, dated to ⁓55 ka by OSL [118]. The site [118] lies within the northern route that promotes east-west dispersals across northern Iran. The site is associated with a fluvial deposit, and environmental and sedimentological analysis suggest the site was occupied during a humid episode within MIS 3 when the rivers draining from the Alborz Mountains were more active than they are today [118, 119].

Though there are fewer UP sites in comparison to the MP, they are also preferentially located in the northern and southern inland routes (Fig 10). These sites are mostly known from caves and rock shelters in the Zagros Mountains [18, 39, 40, 120], with a few open-air sites located in the Iranian Central Plateau [14, 118]. They are associated with various blade-oriented technologies that have been called the Baradostian, the Zagros Aurignacian, the Rostamian and Initial Upper Paleolithic (IUP) [39, 121, 122]. Fossils of *H*. *sapiens* have been reported in a UP context from Eshkaft-e Gavi [123], but there is uncertainty surrounding this UP assignment, owing to the lack of radiometric dates and their unclear stratigraphic positions. A number of radiocarbon dates are available for the UP, and Bayesian modelling of these ages places the onset of the UP and, by inference, the emergence of modern humans in the Zagros region to ⁓45 ka [41]. Although a single radiocarbon date of ⁓49 ka for the UP was also reported from the Kaldar cave in the west-central Zagros [124], this requires some corroboration by other dating methods, lying at the limits of radiocarbon dating. An extensive evaluation of the UP assemblages sites in the Zagros Mountains suggests the transition from the Middle to Upper Paleolithic likely was not a gradual process but rather a rapid movement of *H*. *sapiens* populations from the western and southwestern borders at around 45 ka [18, 125, 126]. In summary, our paleohydrological and archaeological synthesis for MIS 3 (Fig 10) suggests that both Northern Iran and the Zagros Mountains had suitable hydrological conditions to host both resident Neanderthals and incoming *H*. *sapiens* populations. However, the southern inland route is not clearly defined by either the paleoclimate records or the distribution of archaeological sites.

### Hominin occupation and dispersal routes through Iran

Our results have an important bearing on narratives of hominin dispersal in Iran and across the wider region. An absence of stratified LP sites and paucity of dated paleoenvironmental records pre-dating MIS 5 prevent us from discussing this time period in detail. However, in light of comparable evidence from neighboring regions we propose that water availability, raw material resources, and favorable climatic conditions would have been primary drivers of these populations. The lack of temporally well-constrained environmental records and of fossil remains inhibits conclusions, however, tentative proxy and faunal paleontological evidence [61, 77, 81, 82] suggest some humidity prior to MIS 5. It is thus possible that our proposed dispersal routes could also apply to hominin migrations during humid periods prior to MIS 5, however at present this remains unconfirmed.

Much discussion has revolved around ‘southern route’ dispersals of our species during MIS 3, and potentially also MIS 5, and how these facilitated dispersal further east from Iran [13, 18]. Our data synthesis suggests that during MIS 5 much of Iran experienced ameliorated conditions, with the exception of the central deserts. Although the near absence of archaeology directly dated to MIS 5 remains notable, if MIS 5 dispersals through the region occurred, then due to the potential salinity of southern Zagros drainages, and the lack of archaeological sites in coastal regions, it is likely that any ‘southern route’ dispersal was via the interior. Thus there is currently no evidence for the hypothesized coastal dispersal route (route A of Vahdati Nasab et al. [13]). Based on our synthesis, the most likely southern routes for dispersal during MIS 5 was through the interior following the spinal lakes of the Zagros then east across the southern Lut or Jazmurian, rather than the coast.

During MIS 5 there is a considerable amount of evidence for the accessibility of northern routes reaching eastwards from the northern Zagros, traversing the Alborz, either on its north side by the Caspian Sea or along its southern flanks, and then onwards along the Kopet Dagh Mountains and/or the northern Dasht-I Kavir. These northern routes would potentially permit populations to move further into Southwest Asia or facilitate population exchange and interactions between Southwest Asia and Iran. At the end of MIS 5 these connections would then have waned, removing contacts between the Zagros Mountains and areas further east, and isolating Iranian hominin populations. Both the northern and southern inland routes could also have been significant dispersal routes in periods prior to MIS 5. Though there is currently not enough archaeological data to demonstrate them, recent research shows that there was occupation prior to MIS 5. For example Qaleh Kurd cave, situated along the northern route, hosts a Neanderthal tooth dated to ca. 150 ka [26]. Furthermore, our evaluation of climate models suggests repeated opening of the northern routes during all interglacials and some interstadials over the last 300 ka.

There also appears to have been opportunities for dispersal along the northern routes during MIS 3, with evidence for habitable conditions along the southern slopes of the Alborz during this time, where Mirak and a series of open-air sites suggest suitable climatic conditions for habitation in local pluvial phases between ~55–30 ka [110, 118, 119], in agreement with the loess data and paleoclimate modelling. In the Zagros, MP occupations have been dated to between ~77–42 ka, and UP occupations between ~49–25 ka (Fig 10). Thus, an abundance of archaeological sites support a habitable Zagros zone over an extended period of time, even though there is a climatic downturn during MIS 4, indicating that the Zagros may have acted as a refugium during this period.

The fossil and archaeological records may support at least two different groups of MP stone-tool producers in MIS 3 in the Zagros and along the northern routes. The MP assemblages of the Zagros have a Mousterian attribution [25, 127–129], featuring attributes such as a limited use of the Levallois technique, an abundance of heavily retouched scrapers, notched and denticulated pieces and the presence of handaxes, although these are rare and are only found in the vicinity of raw material outcrops [18]. These MP sites are associated with Neanderthal fossils dating to between ⁓70 to ⁓42 ka, indicating that Neanderthals are responsible for the Zagros Mousterian. In contrast, MP sites along the northern routes have yielded an industry similar to the Levantine MP [130, 131]. The sites of Mirak and Qaleh Kurd, though of different ages, are characterized by the common use of the Levallois technique, the abundant presence of points (Levallois or retouched), the lower relative percentage of flake-based heavily retouched scrapers and a lack of handaxes. Though the technology is apparently similar, the Qaleh Kurd archaeological deposit is assigned to Neanderthals based on the recovery of a tooth, but the producers of the Mirak MP assemblages may represent *H*. *sapiens*, as previously suggested for other sites in the Iranian Central Plateau [36]. The latter suggestion for Mirak is based on 3D geometric-morphometric comparison of lithic assemblages between the Mirak MP and UP showing no major difference in flake production technologies [131], as well as their similarity in Levallois production techniques with Levantine assemblages of the same age [118]. Thus, it is possible that the Zagros Mountains was occupied by Neanderthals at the same time that the dispersal of *H*. *sapiens* was occurring across the northern routes.

Neanderthals survived in the west-central Zagros until 42 ka [25], with reported UP sites from along the Zagros placing the onset of the UP, and by inference the initial presence of *H*. *sapiens* at ⁓45 ka [41]. The UP assemblages of the Alborz and northern Dasht-I Kavir are dated to ⁓35 ka in sites such as Mirak and Garm Rud 2. An inter-assemblage comparison of techno-typological features between the UP sites from along the Zagros, such as at Yafteh, Ghar-e Boof, Warwasi and Pebdeh, has suggested high versatility and diversity in blade production technology among the UP populations for approximately the same time range between ⁓42–37 ka [41]. Another important feature which is shared in all UP lithic assemblages in the Zagros, is Arjeneh points, which are absent in sites along the Alborz and northern edge of the Iranian Central Desert [132]. Based on the current data, it appears that Neanderthals and *H*. *sapiens* were both occupying the Zagros, and overlapped for at least ~4 thousand years. The residing *H*. *sapiens* groups were most likely separated populations dispersing from the west and south, with distinct stone tools.

## Conclusions

Our synthesis of paleohydrological, environmental proxy, archaeological and climate modelling data for Iran has suggested that ameliorated hydroclimate conditions supported dispersals across the country during both MIS 5 and MIS 3. Spatio-temporally specific routes through northern Iran have been identified during both these periods, when enhanced moisture led to opportunities to disperse. We find no evidence for a southern coastal route; however, in MIS 5 we identify for the first time a southern inland from the Tigris plains through north-west Iran then along the Zagros and across southern Iran towards Baluchistan.

MIS 5 is a key period for discussions of hominin dispersals, given the documented presence of *H*. *sapiens* to the immediate south-west in the Levant [133] and Arabia [30, 134]. The presence of *H*. *sapiens* further east at this time continues to be a key area of debate in paleoanthropology [135], and some data supports this suggestion in the context of India [31]. This bequeaths Iran a key role in evaluating this issue, lying between the Levant and Arabia, and areas further east such as India. Our data shows much evidence for moisture during MIS 5, with two clear routes for dispersal evidenced, both of which have a preferential concentration of the undated MP sites within them, implying they were used, though there are too few dated sites in Iran to confirm this at present. Importantly there is no environmental barrier to a southern dispersal at this time, supporting suggestions that *H*. *sapiens* expanded through Southwest Asia towards India during MIS 5.

With evidence suggesting relatively widespread humidity in Iran during MIS 5, it is surprising that the only dated MP site is Qaleh Kurd. Given the antiquity of *H*. *sapiens* and Neanderthals, and the presence of earlier hominin fossil sites in surrounding regions [7, 30, 136, 137], the absence of dated sites seems likely to reflect sampling bias. This indicates a key research lacunae that can be readily addressed by concentrating future work on the dispersal routes identified here, then targeting them for archaeological survey and dating. This strategy has worked well in Arabia [33, 35, 48, 49, 134], suggesting that a similar approach will reap dividends in Iran. Notable areas for future survey not only include the northern and southern inland routes, but also areas where little work has been done to date, such as the Lut and Dasht-I Kavir regions and the Sistan Basin.

During MIS 3, proxy and climate model data highlights a potential northern route for dispersal across Iran. This involved traversing the Alborz Mountains, using either the Caspian Sea lowlands, or the southern flanks of the mountains on the margins of the Dasht-I Kavir, or both, then moving onwards along the Kopet Dagh Mountains and then potentially further eastwards into central Asia. This route is broadly temporally coincident with suggestions of *H*. *sapiens* dispersals between 60–48 ka. Both MP and UP site distributions preferentially follow this corridor. Furthermore, the character of lithics along this corridor (both MP and UP) support its use by *H*. *sapiens* during MIS 3. As a consequence, further survey along these northern routes is important for improving our understanding of *H*. *sapiens* dispersals during the Late Pleistocene.

## Supporting information

S1 Table Paleolithic sites evaluated in this study. (PDF)

S2 Table Environmental proxy sites in this study. (PDF)

## References

[1] McMahon H. Recent Survey and Exploration in Seistan. The Geographical Journal. 1906;28(3):209–28. doi: 10.2307/1776729

[2] de Morgan J. Le Plateau iranien pendant l’époque pléistocène: Félix Alcan; 1907.

[3] Garrod DAE. The Palaeolithic of Southern Kurdistan: Excavations in the Caves of Iranian Paleolithic Zarzi and Hazar Merd. Bulletin of the American School of Prehistoric Research. 1930;6:8–43.

[4] Field H, Young MY, Ettinghausen R, Field Museum of Natural H. Contributions to the anthropology of Iran. Chicago: Field Museum Press; 1939.

[5] Ghirshman R. Campagne de fouilles à Suse en 1948–1949. Comptes rendus des séances de l’Académie des Inscriptions et Belles-Lettres. 1949:196–9.

[6] Coon CS. Cave Explorations in Iran: 1949. Pennsylvania: University Museum; 1951.

[7] Solecki RS, editor Shanidar Cave, a Late Pleistocene site in Northern Iraq. Report of the VIth International Congress on the Quaternary; 1964; Warsaw.

[8] Braidwood RJ, Howe B, Reed CA. The Iranian Prehistoric Project. Science. 1961;133(3469):2008–10. 17754651 10.1126/science.133.3469.2008

[9] Hole F, Flannery KV, editors. The prehistory of southwestern Iran: a preliminary report. Proceedings of the Prehistoric Society; 1967: Cambridge University Press.

[10] McBurney C. Paleolithic Excavations in the Zagros Area. Iran. 1970;8:185–86.

[11] Speth J. Kunji Cave. Iran. 1971;9:172–73.

[12] Conard N, Ghasidian E, Heydari-Guran S. The Open-air Late Paleolithic site of Bardia and the Paleolithic Occupation of the Qaleh Gusheh Sand Dunes, Esfahan Province, Iran. In: O M., B F., J J., editors. Iran Paelolithic. Lisbon: International BAR Series; 2009. p. 141–54.

[13] Vahdati Nasab H, Clark GA, Torkamandi S. Late Pleistocene dispersal corridors across the Iranian Plateau: a case study from Mirak, a Middle Paleolithic site on the northern edge of the Iranian Central Desert (Dasht-e Kavir). Quaternary International. 2013;300:267–81.

[14] Vahdati Nasab H, Hashemi M. Playas and Middle Paleolithic settlement of the Iranian Central Desert: The discovery of the Chah-e Jam Middle Paleolithic site. Quaternary International. 2016;408:140–52. doi: 10.1016/j.quaint.2015.11.117

[15] Vahdati Nasab H, Clark G. The upper Paleolithic of the Iranian central desert: The delazian site—A case study. Archaologische Mitteilungen aus Iran und Turan. 2014;46:1–20.

[16] Chevrier B, Berillon G, Asgari Khaneghah A, Antoine P, Bahain J-J, Zeitoun V. Moghanak, Otchounak, Garm Roud 2: nouveaux assemblages paléolithiques dans le Nord de l’Iran. Caractérisations typo-technologiques et attributions chrono-culturelles. Paléorient. 2006;32:59–79. doi: 10.3406/paleo.2006.5190

[17] Vahdati Nasab H, Feiz Z, editors. Paleolithic survey of the northern edge of the Iranian Central Desert between Semnan and Sorkheh. Proceedings of the 12th Annual Iranian Archaeology Conference, pp 465e468 (in Farsi); 2014.

[18] Shoaee MJ, Vahdati Nasab H, Petraglia MD. The Paleolithic of the Iranian Plateau: Hominin occupation history and implications for human dispersals across southern Asia. Journal of Anthropological Archaeology. 2021;62:101–292. doi: 10.1016/j.jaa.2021.101292

[19] Vahdati Nasab H. Paleolithic archaeology in Iran. International Journal of Humanities. 2011;18:63–87.

[20] Biglari F, Shidrang S. The Lower Paleolithic Occupation of Iran. Near Eastern Archaeology. 2006;Vol.69:160–8.

[21] Biglari F, Heydari S, Shidrang S. Ganj Par: The First Evidence for Lower Paleolithic Occupation in the Southern Caspian Basin, Iran. Antiquity. 2004;78.

[22] Biglari F, Abdi K. Palaeolithic artefacts from Cham-e Souran, the Islamabad Plain, Central Western Zagros Mountains, Iran. Archaologische Mitteilungen aus Iran und Turan. 1999;31:1–8.

[23] Dennell R. The Palaeolithic Settlement of Asia. Cambridge: Cambridge University Press; 2008.

[24] Stringer C. The status of Homo heidelbergensis (Schoetensack 1908). Evolutionary Anthropology: Issues, News, and Reviews. 2012;21(3):101–7. doi: 10.1002/evan.21311 22718477

[25] Heydari-Guran S, Benazzi S, Talamo S, Ghasidian E, Hariri N, Oxilia G, et al. The discovery of an in situ Neanderthal remain in the Bawa Yawan Rockshelter, West-Central Zagros Mountains, Kermanshah. PLOS ONE. 2021;16(8):e0253708. doi: 10.1371/journal.pone.0253708 34437543 PMC8389444

[26] Vahdati Nasab H, Berillon G, Hashemi M, Jamet G, Jayez M, Akhavan Kharazian M, et al. Paleolithic Cave of Qaleh Kurd, (Qazvin, IRAN): A Late Pleistocene Occupation with Human Remains. In: Heydari-Guran S, Shirazi R, Ghasidian E, editors. Paleolithic of Iran 1. Archaeological reports. 7. Tehran: Iranian Center for Archaeological Research (ICAR); 2020. p. 22–34.

[27] Zanolli C, Biglari F, Mashkour M, Abdi K, Monchot H, Debue K, et al. A Neanderthal from the Central Western Zagros, Iran. Structural reassessment of the Wezmeh 1 maxillary premolar. Journal of human evolution. 2019;135:102643. doi: 10.1016/j.jhevol.2019.102643 31421316

[28] Mallick S, Li H, Lipson M, Mathieson I, Gymrek M, Racimo F, et al. The Simons Genome Diversity Project: 300 genomes from 142 diverse populations. Nature. 2016;538(7624):201–6. doi: 10.1038/nature18964 27654912 PMC5161557

[29] Mellars P, Gori KC, Carr M, Soares PA, Richards MB. Genetic and archaeological perspectives on the initial modern human colonization of southern Asia. Proceedings of the National Academy of Sciences. 2013;110(26):10699–704. doi: 10.1073/pnas.1306043110 23754394 PMC3696785

[30] Groucutt HS, Grün R, Zalmout IAS, Drake NA, Armitage SJ, Candy I, et al. Homo sapiens in Arabia by 85,000 years ago. Nature Ecology & Evolution. 2018;2(5):800–9. doi: 10.1038/s41559-018-0518-2 29632352 PMC5935238

[31] Blinkhorn J, Achyuthan H, Petraglia M, Ditchfield P. Middle Palaeolithic occupation in the Thar Desert during the Upper Pleistocene: the signature of a modern human exit out of Africa? Quaternary Science Reviews. 2013;77:233–8. doi: 10.1016/j.quascirev.2013.06.012

[32] Armitage SJ, Jasim SA, Marks AE, Parker AG, Usik VI, Uerpmann H-P. The Southern Route “Out of Africa”: Evidence for an Early Expansion of Modern Humans into Arabia. Science. 2011;331(6016):453–6. doi: 10.1126/science.1199113 21273486

[33] Breeze PS, Groucutt HS, Drake NA, Louys J, Scerri EM, Armitage SJ, et al. Prehistory and palaeoenvironments of the western Nefud Desert, Saudi Arabia. Archaeological Research in Asia. 2017;10:1–16.

[34] Delagnes A, Tribolo C, Bertran P, Brenet M, Crassard R, Jaubert J, et al. Inland human settlement in southern Arabia 55,000 years ago. New evidence from the Wadi Surdud Middle Paleolithic site complex, western Yemen. Journal of human evolution. 2012;63(3):452–74. doi: 10.1016/j.jhevol.2012.03.008 22766480

[35] Groucutt HS, White TS, Scerri EML, Andrieux E, Clark-Wilson R, Breeze PS, et al. Multiple hominin dispersals into Southwest Asia over the past 400,000 years. Nature. 2021;597(7876):376–80. doi: 10.1038/s41586-021-03863-y 34471286 PMC8443443

[36] Heydari-Guran S, Ghasidian E. Consistency of the "MIS 5 Humid Corridor Model" for the Dispersal of Early Homo sapiens into the Iranian Plateau. In: Herausgeber*innenkollektiv, editor. Pearls, Politics and Pistachios: Essays in Anthropology and Memories on the Occasion of Susan Pollock’s 65th Birthday. Berlin: ex oriente; 2021. p. 219–38.

[37] Otte M, Shidrang S, Zwyns N, Flas D. New radiocarbon dates for the Zagros Aurignacian from Yafteh cave, Iran. Journal of human evolution. 2011;61(3):340–6. doi: 10.1016/j.jhevol.2011.05.011 21714987

[38] Bérillon G, Asgari Khaneghah A. Garm Roud: une halte de chasse en Iran: paléolithique supérieur. Prigonrieux; [Téhéran]: @rchéo-éditions.com; IFRI; 2016.

[39] Conard N, Ghasidian E. The Rostamian Cultural group and the taxonomy of the Upper Paleolithic in Iran. In: Conard NJ, Drechsler P, Morales A, editors. Between Sand and See. Thubingen: Kerns Verlag; 2011.

[40] Heydari-Guran S, Douka K, Higham T, Münzel SC, Deckers K, Hourshid S, et al. Early upper palaeolithic occupation at Gelimgoush cave, Kermanshah; West-Central Zagros mountains of Iran. Journal of Archaeological Science: Reports. 2021;38. doi: 10.1016/j.jasrep.2021.103050

[41] Shoaee MJ, Vahdati Nasab H, Petraglia M. The First Season of Excavation at Pebdeh Cave in Khuzestan Province. Tehran: 2020.

[42] Zwyns N. The Initial Upper Paleolithic in Central and East Asia: Blade Technology, Cultural Transmission, and Implications for Human Dispersals. Journal of Paleolithic Archaeology. 2021;4(3):19. doi: 10.1007/s41982-021-00085-6

[43] Peel MC, Finlayson BL, McMahon TA. Updated world map of the Köppen-Geiger climate classification. Hydrol Earth Syst Sci. 2007;11(5):1633–44. doi: 10.5194/hess-11-1633-2007

[44] Mehterian S, Pourmand A, Sharifi A, Lahijani HAK, Naderi M, Swart PK. Speleothem records of glacial/interglacial climate from Iran forewarn of future Water Availability in the interior of the Middle East. Quaternary Science Reviews. 2017;164:187–98. doi: 10.1016/j.quascirev.2017.03.028

[45] Heydari-Guran S, Ghasidian E. Late Pleistocene hominin settlement patterns and population dynamics in the Zagros Mountains: Kermanshah region. Archaeological Research in Asia. 2020;21:100161. doi: 10.1016/j.ara.2019.100161

[46] Wang PX, Wang B, Cheng H, Fasullo J, Guo Z, Kiefer T, et al. The global monsoon across time scales: Mechanisms and outstanding issues. Earth-Science Reviews. 2017;174:84–121. doi: 10.1016/j.earscirev.2017.07.006

[47] Liu F, Chai J, Wang B, Liu J, Zhang X, Wang Z. Global monsoon precipitation responses to large volcanic eruptions. Scientific Reports. 2016;6(1):24331. doi: 10.1038/srep24331 27063141 PMC4827032

[48] Breeze PS, Drake NA, Groucutt HS, Parton A, Jennings RP, White TS, et al. Remote sensing and GIS techniques for reconstructing Arabian palaeohydrology and identifying archaeological sites. Quaternary International. 2015;382:98–119. doi: 10.1016/j.quaint.2015.01.022

[49] Breeze PS, Groucutt HS, Drake NA, White TS, Jennings RP, Petraglia MD. Palaeohydrological corridors for hominin dispersals in the Middle East ∼250–70,000 years ago. Quaternary Science Reviews. 2016;144:155–85. doi: 10.1016/j.quascirev.2016.05.012

[50] Drake NA, Lem RE, Armitage SJ, Breeze P, Francke J, El-Hawat AS, et al. Reconstructing palaeoclimate and hydrological fluctuations in the Fezzan Basin (southern Libya) since 130 ka: A catchment-based approach. Quaternary Science Reviews. 2018;200:376–94. doi: 10.1016/j.quascirev.2018.09.042

[51] Lehner B, Verdin K, Jarvis A. New Global Hydrography Derived From Spaceborne Elevation Data. Eos, Transactions American Geophysical Union. 2008;89(10):93–4. doi: 10.1029/2008EO100001

[52] Shimada M, Itoh T, Motooka T, Watanabe M, Shiraishi T, Thapa R, et al. New global forest/non-forest maps from ALOS PALSAR data (2007–2010). Remote Sensing of Environment. 2014;155:13–31. doi: 10.1016/j.rse.2014.04.014

[53] Drake NA, Candy I, Breeze P, Armitage SJ, Gasmi N, Schwenninger JL, et al. Sedimentary and geomorphic evidence of Saharan megalakes: A synthesis. Quaternary Science Reviews. 2022;276:107318. doi: 10.1016/j.quascirev.2021.107318

[54] Allen MB, Mark DF, Kheirkhah M, Barfod D, Emami MH, Saville C. 40Ar/39Ar dating of Quaternary lavas in northwest Iran: constraints on the landscape evolution and incision rates of the Turkish-Iranian plateau. Geophysical Journal International. 2011;185(3):1175–88. doi: 10.1111/j.1365-246X.2011.05022.x

[55] Heidarzadeh G, Ballato P, Hassanzadeh J, Ghassemi MR, Strecker MR. Lake overspill and onset of fluvial incision in the Iranian Plateau: Insights from the Mianeh Basin. Earth and Planetary Science Letters. 2017;469:135–47. doi: 10.1016/j.epsl.2017.04.019

[56] Jones SJ, Arzani N, Allen MB. Tectonic and climatic controls on fan systems: The Kohrud mountain belt, Central Iran. Sedimentary Geology. 2014;302:29–43. doi: 10.1016/j.sedgeo.2013.12.008

[57] Berberian M. Active Faulting and Tectonics of Iran. Zagros Hindu Kush Himalaya Geodynamic Evolution 1981. p. 33–69.

[58] Pekel J-F, Cottam A, Gorelick N, Belward AS. High-resolution mapping of global surface water and its long-term changes. Nature. 2016;540(7633):418–22. doi: 10.1038/nature20584 27926733

[59] Lehner B, Liermann CR, Revenga C, Vörösmarty C, Fekete B, Crouzet P, et al. High-resolution mapping of the world’s reservoirs and dams for sustainable river-flow management. Frontiers in Ecology and the Environment. 2011;9(9):494–502. doi: 10.1890/100125

[60] Otto-Bliesner BL, Rosenbloom N, Stone EJ, McKay NP, Lunt DJ, Brady EC, et al. How warm was the last interglacial? New model–data comparisons. Philosophical Transactions of the Royal Society A: Mathematical, Physical and Engineering Sciences. 2013;371(2001):20130097 doi: 10.1098/rsta.2013.0097 24043870

[61] Krapp M, Beyer RM, Edmundson SL, Valdes PJ, Manica A. A statistics-based reconstruction of high-resolution global terrestrial climate for the last 800,000 years. Scientific Data. 2021;8(1):228. doi: 10.1038/s41597-021-01009-3 34453060 PMC8397735

[62] Beyer RM, Krapp M, Manica A. High-resolution terrestrial climate, bioclimate and vegetation for the last 120,000 years. Scientific Data. 2020;7(1):236. doi: 10.1038/s41597-020-0552-1 32665576 PMC7360617

[63] Djamali M, de Beaulieu j-L, Shah-hosseini M, Andrieu-Ponel V, Ponel P, Amini A, et al. A late Pleistocene long pollen record from Lake Urmia, NW Iran. Quaternary Research. 2008;69(63):413–20. doi: 10.1016/j.yqres.2008.03.004

[64] Litt T, Pickarski N, Heumann G, Stockhecke M, Tzedakis PC. A 600,000 year long continental pollen record from Lake Van, eastern Anatolia (Turkey). Quaternary Science Reviews. 2014;104:30–41. doi: 10.1016/j.quascirev.2014.03.017

[65] McCormack J, Bontognali TRR, Immenhauser A, Kwiecien O. Controls on Cyclic Formation of Quaternary Early Diagenetic Dolomite. Geophysical Research Letters. 2018;45(8):3625–34. doi: 10.1002/2018GL077344

[66] Van Zeist W, Wright HE. Preliminary pollen studies at Lake Zeribar, Zagros mountains, southwestern Iran. Science. 1963;140(3562):65–7. doi: 10.1126/science.140.3562.65 17746007

[67] Stevens LR, Ito E, Schwalb A, Wright HE. Timing of atmospheric precipitation in the Zagros Mountains inferred from a multi-proxy record from Lake Mirabad, Iran. Quaternary research. 2006;66(3):494–500.

[68] Djamali M, Soulié-Märsche I, Esu D, Gliozzi E, Okhravi R. Palaeoenvironment of a Late Quaternary lacustrine–palustrine carbonate complex: Zarand Basin, Saveh, central Iran. Palaeogeography, Palaeoclimatology, Palaeoecology. 2006;237(2):315–34. doi: 10.1016/j.palaeo.2005.12.001

[69] Fattahi M, Walker R. Optical dating of Holocene lake bed sediments of the Nimbluk Plain, Khorasan, Northeast Iran: Implications for the climate change and palaeo-environment. Journal of the Earth and Space Physics. 2015;41(4):1–12.

[70] Sharifi A, Pourmand A, Canuel EA, Ferer-Tyler E, Peterson LC, Aichner B, et al. Abrupt climate variability since the last deglaciation based on a high-resolution, multi-proxy peat record from NW Iran: The hand that rocked the Cradle of Civilization? Quaternary Science Reviews. 2015;123:215–30. doi: 10.1016/j.quascirev.2015.07.006

[71] Karimi A, Frechen M, Khademi H, Kehl M, Jalalian A. Chronostratigraphy of loess deposits in northeast Iran. Quaternary International. 2011;234(1):124–32. doi: 10.1016/j.quaint.2009.08.002

[72] Kehl M, Vlaminck S, Köhler T, Laag C, Rolf C, Tsukamoto S, et al. Pleistocene dynamics of dust accumulation and soil formation in the southern Caspian Lowlands—New insights from the loess-paleosol sequence at Neka-Abelou, northern Iran. Quaternary Science Reviews. 2021;253:106774. doi: 10.1016/j.quascirev.2020.106774

[73] Lauer T, Frechen M, Vlaminck S, Kehl M, Lehndorff E, Shahriari A, et al. Luminescence-chronology of the loess palaeosol sequence Toshan, Northern Iran–A highly resolved climate archive for the last glacial–interglacial cycle. Quaternary International. 2017;429:3–12. doi: 10.1016/j.quaint.2015.03.045

[74] Lauer T, Vlaminck S, Frechen M, Rolf C, Kehl M, Sharifi J, et al. The Agh Band loess-palaeosol sequence–A terrestrial archive for climatic shifts during the last and penultimate glacial–interglacial cycles in a semiarid region in northern Iran. Quaternary International. 2017;429:13–30. doi: 10.1016/j.quaint.2016.01.062

[75] Vlaminck S, Kehl M, Lauer T, Shahriari A, Sharifi J, Eckmeier E, et al. Loess-soil sequence at Toshan (Northern Iran): Insights into late Pleistocene climate change. Quaternary International. 2016;399:122–35.

[76] Blaauw M, Christen JA. Flexible paleoclimate age-depth models using an autoregressive gamma process. Bayesian Analysis. 2011;6(3):457–74, 18.

[77] Ghafarpour A, Khormali F, Meng X, Tazikeh H, Stevens T. Late Pleistocene Climate and Dust Source From the Mobarakabad Loess–Paleosol Sequence, Northern Foothills of the Alborz Mountains, Northern Iran. Frontiers in Earth Science. 2021;9. doi: 10.3389/feart.2021.795826

[78] Carolin SA, Ersek V, Roberts WHG, Walker RT, Henderson GM. Drying in the Middle East During Northern Hemisphere Cold Events of the Early Glacial Period. Geophysical Research Letters. 2019;46(23):14003–10. doi: 10.1029/2019GL084365

[79] Kober F, Zeilinger G, Ivy-Ochs S, Dolati A, Smit J, Kubik PW. Climatic and tectonic control on fluvial and alluvial fan sequence formation in the Central Makran Range, SE-Iran. Global and Planetary Change. 2013;111:133–49. doi: 10.1016/j.gloplacha.2013.09.003

[80] Shabanian E, Siame L, Bellier O, Benedetti L, Abbassi MR. Quaternary slip rates along the northeastern boundary of the Arabia-Eurasia collision zone (Kopeh Dagh Mountains, Northeast Iran). Geophysical Journal International. 2009;178(2):1055–77. doi: 10.1111/j.1365-246X.2009.04183.x

[81] Walker RT, Fattahi M. A framework of Holocene and Late Pleistocene environmental change in eastern Iran inferred from the dating of periods of alluvial fan abandonment, river terracing, and lake deposition. Quaternary Science Reviews. 2011;30(9):1256–71. doi: 10.1016/j.quascirev.2011.03.004

[82] Lisiecki LE, Raymo ME. A Pliocene-Pleistocene stack of 57 globally distributed benthic δ18O records. Paleoceanography. 2005;20(1). doi: 10.1029/2004pa001071

[83] Bayat O, Karimzadeh H, Eghbal MK, Karimi A, Amundson R. Calcic soils as indicators of profound Quaternary climate change in eastern Isfahan, Iran. Geoderma. 2018;315:220–30. doi: 10.1016/j.geoderma.2017.11.007

[84] Hashemi N, Ashouri A, Aliabadian M, Gharaie MM, Marco AS, Louys J, et al. First report of Quaternary mammals from the Qalehjough area, Lut desert, eastern Iran. Palaeontologia Electronica. 2016;19:1–12.

[85] Parsons B, Wright T, Rowe P, Andrews J, Jackson J, Walker R, et al. The 1994 Sefidabeh (eastern Iran) earthquakes revisited: new evidence from satellite radar interferometry and carbonate dating about the growth of an active fold above a blind thrust fault. Geophysical Journal International. 2006;164(1):202–17. doi: 10.1111/j.1365-246X.2005.02655.x

[86] Evenstar LA, Sparks RSJ, Cooper FJ, Lawton MN. Quaternary landscape evolution of the Helmand Basin, Afghanistan: Insights from staircase terraces, deltas, and paleoshorelines using high-resolution remote sensing analysis. Geomorphology. 2018;311:37–50. doi: 10.1016/j.geomorph.2018.03.018

[87] Hamzeh MA, Mahmudy Gharaie MH, Alizadeh Ketek Lahijani H, Djamali M, Moussavi Harami R, Naderi Beni A. Holocene hydrological changes in SE Iran, a key region between Indian Summer Monsoon and Mediterranean winter precipitation zones, as revealed from a lacustrine sequence from Lake Hamoun. Quaternary International. 2016;408:25–39. doi: 10.1016/j.quaint.2015.11.011

[88] Kutzbach J, Chen G, Cheng H, Edwards R, Liu Z. Potential role of winter rainfall in explaining increased moisture in the Mediterranean and Middle East during periods of maximum orbitally-forced insolation seasonality. Climate Dynamics. 2014;42(3):1079–95.

[89] Guagnin M, Breeze P, Shipton C, Ott F, Stewart M, Bateman M, et al. The Holocene humid period in the Nefud Desert: Hunters and herders in the Jebel Oraf palaeolake basin, Saudi Arabia. Journal of Arid Environments. 2020;178:104146. doi: 10.1016/j.jaridenv.2020.104146

[90] Engel M, Brückner H, Pint A, Wellbrock K, Ginau A, Voss P, et al. The early Holocene humid period in NW Saudi Arabia—Sediments, microfossils and palaeo-hydrological modelling. Quaternary International. 2012;266:131–41. doi: 10.1016/j.quaint.2011.04.028

[91] Vaezi A, Ghazban F, Tavakoli V, Routh J, Beni AN, Bianchi TS, et al. A Late Pleistocene-Holocene multi-proxy record of climate variability in the Jazmurian playa, southeastern Iran. Palaeogeography, Palaeoclimatology, Palaeoecology. 2019;514:754–67. doi: 10.1016/j.palaeo.2018.09.026

[92] Petraglia MD, Groucutt HS, Guagnin M, Breeze PS, Boivin N. Human responses to climate and ecosystem change in ancient Arabia. Proceedings of the National Academy of Sciences. 2020;117(15):8263–70. doi: 10.1073/pnas.1920211117 32284422 PMC7165439

[93] Stevens L, Wright H Jr, Ito E. Proposed changes in seasonality of climate during the Lateglacial and Holocene at Lake Zeribar, Iran. The Holocene. 2001;11(6):747–55.

[94] Ghodsi M. Morphometric characteristics of Yardangs in the Lut Desert, Iran. Desert. 2017;22(1):21–9.

[95] Krinsley DB. A Geomorphological and Paleoclimatological Study of the Playas of Iran. Part I. GEOLOGICAL SURVEY RESTON VA, 1970.

[96] Maghsoudi M, Negahban S, Bagheri S. Lut Desert, Barkhan, Shahdad, Sand dunes landforms. Applied Geomorphology of Iran. 2014;2(3):65–78.

[97] Maghsoudi M. Hydro-aeolian Landforms. Desert Landscapes and Landforms of Iran. Cham: Springer International Publishing; 2021. p. 87–98.

[98] Hume GW. The Ladizian: An Industry of the Asian Chopper-Chopping Tool Complex in Iranian Baluchistan: Dorrance; 1976.

[99] Speth JD. Archaeology: The Ladizian: An Industry of the Asian Chopper-Chopping Tool Complex in Iranian Baluchistan. Gary W. Hume. American Anthropologist. 1978;80(3):743-. doi: 10.1525/aa.1978.80.3.02a00850

[100] Field JS, Petraglia MD, Lahr MM. The southern dispersal hypothesis and the South Asian archaeological record: Examination of dispersal routes through GIS analysis. Journal of Anthropological Archaeology. 2007;26(1):88–108. doi: 10.1016/j.jaa.2006.06.001

[101] Oppenheimer S. The great arc of dispersal of modern humans: Africa to Australia. Quaternary International. 2009;202(1):2–13. doi: 10.1016/j.quaint.2008.05.015

[102] Oppenheimer S. A single southern exit of modern humans from Africa: Before or after Toba? Quaternary International. 2012;258:88–99.

[103] Stewart M, Andrieux E, Clark-Wilson R, Vanwezer N, Blinkhorn J, Armitage SJ, et al. Taphonomy of an excavated striped hyena (Hyaena hyaena) den in Arabia: implications for paleoecology and prehistory. Archaeological and Anthropological Sciences. 2021;13(8):139. doi: 10.1007/s12520-021-01365-6

[104] Jennings RP, Parton A, Clark-Balzan L, White TS, Groucutt HS, Breeze PS, et al. Human occupation of the northern Arabian interior during early Marine Isotope Stage 3. Journal of Quaternary Science. 2016;31(8):e2920. doi: 10.1002/jqs.2920

[105] Naderi M, Raeisi E, Zarei M. The impact of halite dissolution of salt diapirs on surface and ground water under climate change, South-Central Iran. Environmental Earth Sciences. 2016;75(8):708.

[106] Pollastro R, Persits F, Steinshouer DW, cartographers. Surficial geology of Iran: U.S. Geological Survey; 1999.

[107] Roach NT, Hatala KG, Ostrofsky KR, Villmoare B, Reeves JS, Du A, et al. Pleistocene footprints show intensive use of lake margin habitats by Homo erectus groups. Scientific Reports. 2016;6(1):1–9. 27199261 10.1038/srep26374 PMC4873780

[108] Jennings RP, Shipton C, Breeze P, Cuthbertson P, Bernal MA, Wedage WO, et al. Multi-scale Acheulean landscape survey in the Arabian Desert. Quaternary International. 2015;382:58–81.

[109] Rosenfeld A, Nathan Y, Feibel C, Schilman B, Halicz L, Goren-Inbar N, et al. Palaeoenvironment of the Acheulian Gesher Benot Ya’aqov Pleistocene lacustrine strata, Northern Israel––lithology, ostracod assemblages and ostracod shell geochemistry. Journal of African Earth Sciences. 2004;38(2):169–81.

[110] Kelly RL. The lifeways of hunter-gatherers: the foraging spectrum: Cambridge University Press; 2014.

[111] Trinkaus E, Biglari F. Middle Paleolithic Human Remains from Bisitun Cave, Iran. Paléorient. 2006;Vol.32:105–11. doi: 10.2307/41496783

[112] Bahraminia M, Niknami KA, Khosrowzadeh A, Nymark A. High altitude Middle Palaeolithic open-air locales of the Miankouh, Thrust Zagros Mountains, Iran. Journal of Archaeological Science: Reports. 2022;44:103537. doi: 10.1016/j.jasrep.2022.103537

[113] Heydari-Guran S, Ghasidian E. The MUP Zagros Project: tracking the Middle–Upper Palaeolithic transition in the Kermanshah region, west-central Zagros, Iran. Antiquity. 2017;91(355):e2. Epub 2017/01/20. doi: 10.15184/aqy.2016.261

[114] Heydari-Guran S, Ghasidian E, Conard NJ. Middle Paleolithic Settlement on the Iranian Central Plateau. In: Conard NJ, Delagnes A, editors. Settlement Dynamics of the Middle Paleolithic and Middle Stone Age. IV. Tübingen: Kerns Verlag; 2015. p. 171–203.

[115] Biglari F. The Preliminary Survey of Paleolithic Sites in the Kashan Region. In: Shahmirzadi SM, editor. The Silversmiths of Sialk (Sialk Reconsideration Project)(in Farsi). Tehran: Archaeological Research Center; 2004. p. 151–68.

[116] Heydari-Guran S, Ghasidian E. Palaeolithic survey in the Arisman region, western Central Iranian Plateau. In: Vatandoust A, Parzinger H, Helwing B, editors. n Early Mining and Metallurgy on the Western Central Iranian Plateau: The First Five Years of Work. 9. Mainz: Philipp von Zabern; 2011. p. 484–98.

[117] Heydari M, Guérin G, Zeidi M, Conard NJ. Bayesian luminescence dating at Ghār-e Boof, Iran, provides a new chronology for Middle and Upper Paleolithic in the southern Zagros. Journal of human evolution. 2021;151:102926. doi: 10.1016/j.jhevol.2020.102926 33429259

[118] Vahdati Nasab H, Berillon G, Jamet G, Hashemi M, Jayez M, Khaksar S, et al. The open-air Paleolithic site of Mirak, northern edge of the Iranian Central Desert (Semnan, Iran): Evidence of repeated human occupations during the late Pleistocene. Comptes Rendus Palevol. 2019;18(4):465–78. doi: 10.1016/j.crpv.2019.02.005

[119] Akhavan Kharazian M, Jamet G, Puaud S, Vahdati Nasab H, Hashemi M, Guerin G, et al. First geoarchaeological study of a Palaeolithic site on the northern edge of the Iranian Central Desert: Mirak (Semnan, Iran). Journal of Arid Environments. 2022;201:104739. doi: 10.1016/j.jaridenv.2022.104739

[120] Otte M, Biglari F, Flas D, Shidrang S, Zwyns N, Mashkour M, et al. The Aurignacian in the Zagros region: new research at Yafteh Cave, Lorestan, Iran. Antiquity. 2007;81(311):82–96. Epub 2015/01/02. doi: 10.1017/S0003598X00094850

[121] Solecki RS. The Baradostian industry and the upper palaeolithic in the Near East. New York 1958.

[122] Olszewski DI, Dibble HL. The Zagros Aurignacian. Current Anthropology. 1994;35(1):68–75.

[123] Scott JE, Marean CW. Paleolithic hominin remains from Eshkaft-e Gavi (southern Zagros Mountains, Iran): description, affinities, and evidence for butchery. Journal of human evolution. 2009;57(3):248–59. doi: 10.1016/j.jhevol.2009.05.012 19660782

[124] Bazgir B, Ollé A, Tumung L, Becerra-Valdivia L, Douka K, Higham T, et al. Understanding the emergence of modern humans and the disappearance of Neanderthals: Insights from Kaldar Cave (Khorramabad Valley, Western Iran). Scientific Reports. 2017;7:43460. doi: 10.1038/srep43460 https://www.nature.com/articles/srep43460#supplementary-information. 28252042 PMC5333163

[125] Shidrang S. The early Upper Paleolithic of Zagros: techno-typological assessment of three Baradostian lithic assemblages from Khar Cave (Ghar-e Khar), Yafteh Cave and Pa-Sangar Rockshelter in the Central Zagros, Iran 2015.

[126] Ghasidian E. Rethinking the Upper Paleolithic of the Zagros Mountains. PaleoAnthropology. 2019:240–310.

[127] Biglari F, Heydari S. Do-Ashkaft: a recently discovered Mousterian cave site in the Kermanshah Plain, Iran. Antiquity. 2001;75(289):487–8. Epub 2015/01/02. doi: 10.1017/S0003598X00088578

[128] Dibble HL. The mousterian industry from Bisitun cave (Iran). Paléorient. 1984:23–34.

[129] Lindly JM. The Zagros Mousterian: a regional perspective. Tempe: Arizona State University; 2005.

[130] Shea JJ. Sink the Mousterian? Named stone tool industries (NASTIES) as obstacles to investigating hominin evolutionary relationships in the Later Middle Paleolithic Levant. Quaternary International. 2014;350:169–79. doi: 10.1016/j.quaint.2014.01.024

[131] Hashemi SM, Vahdati Nasab H, Berillon G, Oryat M. An investigation of the flake-based lithic tool morphology using 3D geometric morphometrics: A case study from the Mirak Paleolithic Site, Iran. Journal of Archaeological Science: Reports. 2021;37:102948. doi: 10.1016/j.jasrep.2021.102948

[132] Abolfathi M, Baills H, Bonilauri S, Forestier H, Vahdati Nasab H, Khaneghah A, et al. Recent history of researches on Upper Paleolithic in Zagros and Alborz (Iran). L Anthropologie. 2019;vol 122:749–61.

[133] Valladas H, Reyss J-L, Joron J-L, Valladas G, Bar-Yosef O, Vandermeersch B. Thermoluminescence dating of Mousterian Troto-Cro-Magnon’remains from Israel and the origin of modern man. Nature. 1988;331(6157):614–6.

[134] Stewart M, Clark-Wilson R, Breeze PS, Janulis K, Candy I, Armitage SJ, et al. Human footprints provide snapshot of last interglacial ecology in the Arabian interior. Science Advances. 2020;6(38):eaba8940. doi: 10.1126/sciadv.aba8940 32948582 PMC7500939

[135] Groucutt HS, Petraglia MD, Bailey G, Scerri EML, Parton A, Clark-Balzan L, et al. Rethinking the dispersal of Homo sapiens out of Africa. Evolutionary Anthropology: Issues, News, and Reviews. 2015;24(4):149–64. doi: 10.1002/evan.21455 26267436 PMC6715448

[136] Hershkovitz I, Weber GW, Quam R, Duval M, Grün R, Kinsley L, et al. The earliest modern humans outside Africa. Science. 2018;359(6374):456–9. doi: 10.1126/science.aap8369 29371468

[137] Grün R, Stringer C, McDermott F, Nathan R, Porat N, Robertson S, et al. U-series and ESR analyses of bones and teeth relating to the human burials from Skhul. Journal of human evolution. 2005;49(3):316–34. doi: 10.1016/j.jhevol.2005.04.006 15970310

