# Supplementary material for: Defining paleoclimatic routes and opportunities for hominin dispersals across Iran
Source: PLoS One. 2023 Mar 1;18(3):e0281872. doi: 10.1371/journal.pone.0281872 (PMC9977010; doi:10.1371/journal.pone.0281872)
Supplement: S1 Table — (PDF) [file pone.0281872.s001.pdf]

SI 1 – ARCHAEOLOGICAL DATABASE (AFTER SHOAEE, VAHDATI NASAB, & PETRAGLIA, 2021)

Site Name	Lat	Long	Type (Cave, Shelter or Open Air)	Typology (LP, MP, UP)	Dating method	Mean (ka) - directly dated assemblage	References	Coordinate Source	Location confidence	Stratified (?)	Distance to water (m)
**Maragheh**	37 26	46 15	C	LP	comparative	null	Sadek-Kooros 1976	geo-ref	low	N	27110
**Daroungar**	37 28	59 04	S	LP	comparative	null	Sadraei et al. 2018	reference	high	N	135
**Tamtameh**	37 29	44 44	C	MP	comparative	null	Coon 1957	Yousefi et al. 2020	low	N	13908
**Kiaram I**	37 15	55 39	C	MP	comparative	null	McBurney 1964		low	N	11902
**Tabarak**	37 08	58 34	S	LP	comparative	null	Sadraei et al. 2019a	geo-ref	med	N	9120
**Ganj Par**	36 53	49 27	S	LP	comparative	null	Biglari et al. 2004	reference	low	N	23841
**Darband**	36 50	49 39	C	LP	comparative	null	Biglari and Shidrang 2006	reference	low	N	24650
**Khal Vasht**	36 46	49 44	S	UP	comparative	null	Biglari and Abdi 2000	reference	low	N	14836
**Shiwatoo**	36 45	45 38	S	LP	comparative	null	Jaubert et al. 2006	reference	low	N	108
**Band-e Pay**	36 35	51 36	S	MP	comparative	null	Vahdati Nasab et al. 2017	reference	high	N	19185
**Chal Tapeh**	36 35	47 14	S	MP	comparative	null	Heydari-Guran 2014	Yousefi et al. 2020	low	N	23362
**Garm Roud 2**	36 21	52 25	OA	UP	C14	29-23 cal BP	Antoine et al. 2016; Berillon et al. 2007; Chevrier et al. 2006	reference	high	N	5505
**Damghani**	36 11	57 42	S	MP	comparative	null	Sadraei et al. 2017	reference	high	N	4108
**Tepe Khaleseh**	36 09	49 09	S	LP	comparative	null	Alibaigi and Khosravi 2009	reference	low	N	5442
**Chah-e Jam**	36 01	54 24	S	MP-UP	comparative	null	Vahdati Nasab and Hashemi 2016	reference	high	N	0
**Nargeh**	36 00	49 35	S	MP	comparative	null	Biglari 2003	reference	low	N	2849
**Sorheh**	35 59	50 57	RS	MP	comparative	null	Hariryan et al. 2021	Hariryan et al. 2021	high	Y	11816
**Qaleh Kurd**	35 47	48 51	C	MP-UP	C14-ESR	null	Vahdati Nasab et al. in prep.	reference	high	Y	11970
**Sar Darreh**	35 47	48 51	S	MP-UP	comparative	null	Nateqi et al. 2021	reference	high	N	11970

**Moghanak**	35 35	52 15	S	MP	comparative	null	Chevrier et al. 2006; Berillon et al. 2007	reference	high	N	15800
**Ochunak**	35 35	52 14	S	MP	comparative	null	Chevrier et al. 2006; Berillon et al. 2007	reference	high	N	14768
**Delazian**	35 29	53 26	S	UP	comparative	null	Vahdati Nasab and Clark 2014	reference	high	N	4380
**Mirak 8**	35 28	53 25	OA	MP-UP	OSL	21-28<br>26-33<br>43-55	Vahdati Nasab et al. 2019; Heydari et al. 2020	reference	high	Y	3412
**Soufi Abad**	35 27	53 19	S	MP-UP	comparative	null	Vahdati Nasab and Feiz 2014	reference	high	N	12130
**Sepid Dasht**	35 23	49 36	S	MP	comparative	null	Vahdati Nasab et al. 2009	reference	high	N	36212
**Zaviyeh**	35 22	50 31	S	MP-UP	comparative	null	Heydari-Guran et al. 2014	reference	high	N	6419
**ZA 16**	35 22	50 32	S	MP	comparative	null	Heydari-Guran 2014	Yousefi et al. 2020	low	N	5625
**Shour Qazi**	35 16	52 08	S	MP-UP	comparative	null	Nateqi et al. 2021	reference	high	N	40209
**Kashafrud**	35 12	61 03	S	LP	comparative	null	Ariai and Thibault 1975; Jamialahmadi et al. 2008	reference	med	N	4202
**Gelimogoush**	34 41	46 52	RS	UP	C14	42-33 Cal BP	Heydari et al. 2021	reference	high	Y	11010
**Bawa Yawan**	34 37	46 55	RS	MP-UP	C14	44-34 cal BP	Heydari-Guran 2021 a	reference	high	N	3081
**Ashkaft-e Baluch**	34 34	46 58	C	MP	comparative	null	Heydari-Guran 2020	Yousefi et al. 2020	low	N	2495
**Ashkaft-e Dariwa**	34 27	46 10	RS	MP	comparative	null	Heydari-Guran 2020	Yousefi et al. 2020	low	N	20301
**Bisitun**	34 23	47 25	C	MP-UP	comparative	null	Coon 1951; Dibble 1984	geo-ref	low	N	1495
**Do eshkaft**	34 24	47 07	C	MP	comparative	null	Biglari and Heydari 2001	geo-ref	low	N	4874
**Ghar-I Khar**	34 23	47 26	C	MP-UP	comparative	null	Smith 1986, 1967; Shidrang et al. 2016	geo-ref	low	N	1841
**Ghobeh**	34 23	47 07	C	MP	comparative	null	Braidwood and Howe 1960;	geo-ref	low	N	4556

							Braidwood et al. 1961; Lindly 2005				
**Mar Aftab**	34 23	47 26	C	MP	comparative	null	Jaubert et al. 2006, 2009	reference	low	N	1225
**Mar Do Dar**	34 23	47 26	C	MP-UP	comparative	null	Jaubert et al. 2006, 2009	reference	low	N	1225
**Mar Tarik**	34 23	47 26	C	MP	comparative	null	Jaubert et al. 2006, 2009	reference	low	N	1225
**Warwasi**	34 25	47 11	RS	MP-UP	comparative	null	Braidwood and Howe 1960; Dibble and Holdaway 1993; Olszewski 1993	geo-ref	low	N	11896
**Ashkaft-e Cham-e Emam Hasan**	34 22	45 44	RS	MP	comparative	null	Heydari-Guran 2020	Yousefi et al. 2020	low	N	11455
**Gakia**	34 16	47 12	S	LP-MP	comparative	null	Braidwood 1960	geo-ref	low	N	45
**Geleh**	34 04	51 16	S	LP	comparative	null	Biglari 2004	geo-ref	low	N	10112
**Wezmeh**	34 03	46 38	C	UP	C14/U-Th/γ spec	25-20<br>70-63?<br>43-11?<br>19.9-19.7 cal BP<br>13.1-12.4 cal BP	Djamali et al. 2011; Mashkour et al. 2009; Trinkaus et al. 2008	reference	low	N	8129
**Khezri**	34 02	58 49	S	MP	comparative	null	Barfi et al. 2013	reference	low	N	25328
**Kaftar Kouh**	34 00	58 11	S	LP-MP	comparative	null	Sadraei et al. 2019b	geo-ref	high	N	3234
**Niasar**	33 57	51 08	S	MP	comparative	null	Heydari-Guran 2014	Yousefi et al. 2020	low	N	9491
**Sefid Ab**	33 57	51 21	S	UP	comparative	null	Shidrang 2009	paper map	low	N	3648
**Cheshmaehsangi 4**	33 55	46 42	S	MP	comparative	null	Heydari-Guran 2020	Yousefi et al. 2020	low	N	4619
**Kaftarkhun**	33 53	51 22	S	MP	comparative	null	Heydari-Guran 2014	Yousefi et al. 2020	low	N	11087
**Qaleh Gousheh (Bardia)**	33 45	51 47	S	MP-UP	comparative	null	Conard et al., 2009	reference	low	N	24354
**Koran Bozan**	33 44	47 21	S	LP-MP	comparative	null	Alibaigi and Niknami 2004; Alibaigi et al. 2011	reference	low	N	953
**Pal Barik**	33 45	47 03	S	LP-MP	comparative	null	Mortensen 1993	geo-ref	low	N	5333
**Ghar Huchi**	33 45	47 05	C	MP	comparative	null	Mortensen 1993	Yousefi et al. 2020	low	N	4532

**Humian**	33 40	47 44	RS	MP	U/TH	148,000 ±35,000	McBurney 1970; Bewley 1984; Skinner 1965	geo-ref	low	N	5912
**Bard Spid**	33 38	47 36	RS	MP	comparative	null	McBurney 1970	Yousefi et al. 2020	low	N	19107
**QG8**	33 39	52 06	RS	MP	comparative	null	Heydari-Guran 2014	Yousefi et al. 2020	low	N	4676
**QG9**	33 40	52 10	RS	MP	comparative	null	Heydari-Guran 2014	Yousefi et al. 2020	low	N	3846
**Mar Gwergalan**	33 33	47 06	C	LP-UP	comparative	null	Mortensen 1993; Davoudi et al. 2015	reference	low	N	8718
**Pa Sangar**	33 33	48 21	RS	UP	comparative	null	Hole and Flannery 1967	geo-ref	low	N	15705
**Yafteh**	33 34	48 16	C	UP	C14	>40-30.1 calBP	Hole and Flannery 1967; Otte et al. 2011	reference	low	N	13185
**Kalat-e Shour**	33 35	58 53	S	MP	comparative	null	Sadraei and Anani 2018	geo-ref	med	N	18510
**Hol Abad I**	33 34	52 00	RS	MP	comparative	null	Heydari-Guran 2014	Yousefi et al. 2020	low	N	11733
**Ghamari**	33 29	48 20	C	MP-UP	comparative	null	Hole and Flannery 1967; Bazgir et al. 2014	reference	high	N	8958
**Kaldar**	33 33	48 17	C	MP-UP	AMS-TL	26-23 47-43	Becerra-Valdivia et al. 2017; Bazgir et al. 2014, 2017	reference	high	Y	12743
**Sorkhe Lizeh**	33 31	48 13	C	UP	comparative	null	Roustaei et al. 2002, 2004	reference	low	N	6762
**Takht-e Shir**	33 30	48 14	C	UP	comparative	null	Roustaei et al. 2002, 2004	reference	low	N	6739
**Gar Arjeneh**	33 26	48 20	RS	MP-UP	comparative	null	Hole and Flannery 1967; Roustaei et al. 2004; Bazgir et al. 2014	reference	high	Y	5700
**Gilvaran**	33 28	48 18	C	MP-UP	comparative	null	Roustaei et al. 2004; Bazgir et al. 2014	reference	high	Y	4743
**Kunji**	33 26	48 21	C	MP	C14	>40	Hole and Flannery 1967; Baumler and Speth 1993	geo-ref	low	N	7882

**Amar Merdeg**	33 14	46 14	S	LP	comparative	null	Biglari et al. 2000; Darabi et al. 2012	reference	low	N	0
**Khunik**	32 40	60 05	C	MP	comparative	null	Coon 1951	geo-ref	low	N	13922
**Bar Andaz**	32 32	59 49	S	MP	comparative	null	Nikzad et al. 2015	reference	low	N	7335
**Koohrang**	32 28	50 07	S	MP	comparative	null	Roustaei 2010	reference	low	N	25794
**Chehel Dokhtaran**	32 28	59 35	S	MP	comparative	null	Barfi and Soroush 2013	reference	low	N	22381
**Pebdeh**	32 26	49 14	C	IUP	C14	42-36 cal BP	Shoaei et al. in prep.	reference	high	?	13958
**Qaleh Bozi**	32 24	51 31	C	MP	comparative	null	Biglari et al. 2009	reference	low	N	6408
**Farsan**	32 15	50 35	S	MP	comparative	null	Nikzad et al. 2015	reference	low	N	19205
**Khvanand**	32 18	59 08	S	MP	comparative	null	Nikzad et al. 2015	reference	low	N	15989
**Kul Farah**	31 52	49 56	RS	UP	comparative	null	Barfi 2009	reference	high	N	9318
**Basht**	30 22	51 09	C-RS	MP-UP	comparative	null	Ghasidian et al. 2009	reference	high	N	11206
**Khan Ahmad**	30 19	51 05	C-RS	UP	comparative	null	Ghasidian et al. 2009	reference	med	N	13156
**Soukhteh**	30 20	51 01	C-RS	UP	comparative	null	Ghasidian et al. 2009	reference	low	N	15763
**Shiv**	30 22	51 18	S	MP	comparative	null	Heydari-Guran 2014	Yousefi et al. 2020	low	N	11431
**Ghar-I Boof**	30 17	51 26	C	UP	C14	41.3-35 cal BP	Conard and Ghasidian 2011; Ghasidian 2014; Becerra-Valdivia et al. 2017	reference	high	Y	5247
**Yagheh Sangar**	30 16	51 26	C-RS	UP	comparative	null	Ghasidian et al. 2009	reference	low	N	4206
**Borz**	30 16	51 27	RS	MP	comparative	null	Heydari-Guran 2014	Yousefi et al. 2020	low	N	4276
**BZC**	30 04	52 43	C	MP	comparative	null	Rosenberg 1990	Yousefi et al. 2020	low	N	17472
**BAC**	30 05	52 24	C	MP	comparative	null	Rosenberg 1991	Yousefi et al. 2020	low	N	12465
**BBC**	30 05	52 24	C	MP	comparative	null	Rosenberg 1992	Yousefi et al. 2020	low	N	12465
**Eshkaft-e Gavi**	29 54	52 41	C	UP	C14	>28<br>24<br>19	Rosenberg 1985, 2003	reference	low	Y	5989
**KZC**	29 56	52 38	C	MP	comparative	null	Rosenberg 1989	Yousefi et al. 2020	low	N	6351
**Momen Abad**	29 55	56 27	S	UP	comparative	null	ICAR Reports*	reference	high	N	9069

**Arsanjan**	29 53	53 20	C-RS-S	MP	comparative	null	Ikeda 1979; Tsuneki et al. 2012	reference	low	N	14208
**Pariz (Koho Cave)**	29 48	55 43	C	MP	comparative	null	Torkamandi and Khodabakhshi Parizi 2018	reference	high	N	21866
**Pariz (Deh Ayaz)**	29 49	55 42	S	MP	comparative	null	Torkamandi and Khodabakhshi Parizi 2018	reference	low	N	20735
**LPC**	29 48	52 37	RS	MP	comparative	null	Rosenberg 1988	Yousefi et al. 2020	low	N	7216
**Helak**	29 32	51 50	C	UP	comparative	null	Vahdati Nasab et al. 2008	reference	low	N	37341
**Ghadi Barmi Shour**	29 26	52 40	C	UP	comparative	null	Piperno 1974	geo-ref	low	N	4761
**KRMS-04**	29 00	58 52	S	LP-MP	comparative	null	Zeidi et al. 2021	geo-ref	low	N	5937
**KRMS-05**	29 02	58 50	S	LP-MP	comparative	null	Zeidi et al. 2021	geo-ref	low	N	8275
**KRMS-06**	29 00	59 00	S	LP-MP	comparative	null	Zeidi et al. 2021	geo-ref	low	N	2750
**KRMS-07**	29 02	58 58	S	LP-MP	comparative	null	Zeidi et al. 2021	geo-ref	low	N	867
**KRMS-02**	28 57	58 46	S	LP-MP	comparative	null	Zeidi et al. 2021	geo-ref	low	N	139
**KRMS-08**	28 58	59 19	S	LP-MP	comparative	null	Zeidi et al. 2021	geo-ref	low	N	258
**KRMS-09**	28 57	59 21	S	LP-MP	comparative	null	Zeidi et al. 2021	geo-ref	low	N	139
**KRMS-10**	28 56	59 20	S	LP-MP	comparative	null	Zeidi et al. 2021	geo-ref	low	N	894
**KRMS-03**	28 56	58 51	S	LP-MP	comparative	null	Zeidi et al. 2021	geo-ref	low	N	3116
**KRMS-01**	28 39	58 31	S	LP-MP	comparative	null	Zeidi et al. 2021	geo-ref	low	N	2246
**Jahrom**	28 33	53 42	S	MP	comparative	null	Piperno 1972	Yousefi et al. 2020	low	N	34891
**Panj Angusht**	28 12	61 10	S	LP-MP	comparative	null	Mosapour Negari et al. 2020	reference	low	N	18798
**Simish**	27 23	62 17	S	LP	comparative	null	Hume 1976; Vahdati Nasab et al. 2010	geo-ref	low	N	0
**Pole Mashkid**	27 09	62 46	S	LP	comparative	null	ICAR Reports*	reference	high	N	8645
**Ladiz**	27 05	61 58	S	LP	comparative	null	Hume 1976; Vahdati Nasab et al. 2010	geo-ref	low	N	1305
**Mashkid**	27 04	61 55	S	LP	comparative	null	Hume 1976; Vahdati Nasab et al. 2010	geo-ref	low	N	502
**Qeshm**	26 49	55 47	S	MP	comparative	null	Dashtizadeh 2010	reference	low	N	0
**Patanag**	26 32	61 34	RS	UP	comparative	null	ICAR Reports*	reference	high	N	32506
**Toolgi Hawr**	26 30	61 39	C	UP	comparative	null	ICAR Reports*	reference	high	N	30976

**Ziarat Godan IV**	25 39	60 07	S	LP	comparative	null	ICAR Reports*	reference	high	N	545
**Angoor Abad**	25 36	60 07	S	LP	comparative	null	ICAR Reports*	reference	high	N	2205
**Kooh Pzm II**	25 21	60 20	S	LP	comparative	null	ICAR Reports*	reference	high	N	315
**Kooh Pzm III**	25 21	60 20	S	LP	comparative	null	ICAR Reports*	reference	high	N	542

***Iranian Center for Archaeological Research**

<u>**Archaeological database references**</u>

Alibaigi, S and S. Khosravi. 2009. Tepeh Khaleseh: a new Neolithic and Palaeolithic site in the Abharrud basin in north-western Iran. Antiquity 83, issue 319. Project Gallery.

Alibaigi, S, et al. 2011. Palaeolithic open-air sites revealed in the Kuran Buzan Valley, Central Zagros, Iran. Antiquity. Volume 85, issue 329, project gallery.

Alibaigi, S., and K. Niknami. 2014. Archaeological Investigations of Kuran Buzan Valley in Central Zagros. Pazhohesh-haye Bastanshenasi Iran 4(6): 7-26.

Antoine, P., Bahain, J.-J., Ghaleb, B., Mercier, N., 2016. The Chronostratigraphic Framework at Garm Roud, in: Bérillon, G., Asgari Khaneghah, A. (Eds.), Garm Roud : une halte de chasse en Iran : paléolithique supérieur. @rchéo-éditions.com ; IFRI, Prigonrieux; [Téhéran].

Ariai, A., Thibault, C., 1975. Nouvelles précisions à propos de l'outillage paléolithique ancien sur galets du Khorassan (Iran). Paléorient, 101-108.

Barfi, C., 2009. Report of the Paleolithic lithic findings at Kul Farah, Izeh. Bastanshenasi va Tarikh 23(2): 10-14 (in Farsi)

Barfi, C., and M.-R. Soroush. 2013. A Middle Paleolithic Period Site in Sarbisheh, South Khorasan Province. Proceedings of the 1st National Conference on Archaeology of Iran, University of Birjand. (in Farsi).

Barfi, C., Zaferanlou, R., and M.-R. Soroush. 2013. an open-air Paleolithic site in Qaenat, South Khorasan Province. Proceedings of the 1st National Conference on Archaeology of Iran, University of Birjand. (in Farsi).

Baumler, M.F., Speth, J.D., 1993. A Middle Paleolithic assemblage from kunji cave, Iran. The Paleolithic prehistory of the zagros-Taurus, 1-73.

Bazgir, B., Ollé, A., Tumung, L., Becerra-Valdivia, L., Douka, K., Higham, T., van der Made, J., Picin, A., Saladié, P., López-García, J.M., Blain, H.-A., Allué, E., Fernández-García, M., Rey-Rodríguez, I., Arceredillo, D., Bahrololoumi, F., Azimi, M., Otte, M., Carbonell, E., 2017. Understanding the emergence of modern humans and the disappearance of Neanderthals: Insights from Kaldar Cave (Khorramabad Valley, Western Iran). Scientific Reports 7, 43460.

Bazgir, B., Otte, M., Tumung, L., Ollé, A., Deo, S.G., Joglekar, P., López-García, J.M., Picin, A., Davoudi, D., van der Made, J., 2014. Test excavations and initial results at the Middle and Upper Paleolithic sites of Gilvaran, Kaldar, Ghamari caves and Gar Arjene Rockshelter, Khorramabad Valley, western Iran. Comptes Rendus Palevol 13, 511-525.

Becerra-Valdivia, L., Douka, K., Comeskey, D., Bazgir, B., Conard, N.J., Marean, C.W., Ollé, A., Otte, M., Tumung, L., Zeidi, M., Higham, T.F.G., 2017. Chronometric investigations of the Middle to Upper Paleolithic transition in the Zagros Mountains using AMS radiocarbon dating and Bayesian age modelling. Journal of Human Evolution 109, 57-69.

Berillon, G., Asgari Khaneghah, A., Antoine, P., Bahain, J.-J., Chevrier, B., Zeitoun, V., Aminzadeh, N., Beheshti, M., Chanzanagh, H., Nochadi, S., 2007. Discovery of new open-air Paleolithic localities in Central Alborz, Northern Iran. Journal of human evolution 52, 380-387.

Bewley, R.H., Levine, M., Leroi-Gourhan, A., Green, C., 1984. The Cambridge University Archaeological Expedition to Iran 1969. Iran 22, 1-38.

Biglari, F, and S. Heydari-Guran. 2001. Do-Ashkaft: a Recently Discovered Mousterian Cave Site in the Kermanshah Plain, Iran. Antiquity. 75: 487-488.

Biglari, F., 2003. Nargeh: a Middle Paleolithic site in Qazvin Plain. Gozaresh-haye Bastanshenasi 2: 165-171.

Biglari, F., 2004. The Preliminary Survey of Paleolithic Sites in the Kashan Region, in: Shahmirzadi, S.M. (Ed.), The Silversmiths of Sialk (Sialk Reconsideration Project)(in Farsi). Archaeological Research Center, Tehran, pp. 151-168.

Biglari, F., and H. Abdi. 2000. Preliminary Report of the survey at Khalvasht Rockshelter, Amarlou, Guilan. Bastanshenasi va Tarikh 15(1, 2): 72-68.

Biglari, F., Heydari, S., Shidrang, S., 2004. Ganj Par: The First Evidence for Lower Paleolithic Occupation in the Southern Caspian Basin, Iran. Antiquity 78.

Biglari, F., Javeri, M., Mashkour, M., Yazdi, M., Shidrang, S., Tengberg, M., Taheri, K., Darvish, J., 2009. Test excavations at the Middle Paleolithic sites of Qaleh Bozi, Southwest of Central Iran, A preliminary report, in: M., O., F., B., J., J. (Eds.), International Union for Prehistoric and Protohistoric Science, Lisbon.

Biglari, F., Nokandeh, G., Heydari, S., 2000. A recent find of a possible Lower Palaeolithic assemblage from the foothills of the Zagros Mountains. Antiquity 74, 749-750.

Biglari, F., Shidrang, S., 2006. The Lower Paleolithic Occupation of Iran. Near Eastern Archaeology Vol.69, 160-168.

Braidwood, R.J. & R. Howe. 1960. Prehistoric investigations in Iraqi Kurdistan (Oriental Institute Studies 31). Chicago: University of Chicago Press.

Braidwood, R.J., 1960. Seeking the World's First Farmers in Persian Kurdistan. The Illustrated London News 237, 695–697.

Braidwood, R.J., Howe, B., Reed, C.A., 1961. The Iranian Prehistoric Project. Science 133, 2008-2010.

Chevrier, B., Berillon, G., Asgari Khaneghah, A., Antoine, P., Bahain, J.-J., Zeitoun, V., 2006. Moghanak, Otchounak, Garm Roud 2 : nouveaux assemblages paléolithiques dans le Nord de l'Iran. Caractérisations typo-technologiques et attributions chrono-culturelles. Paléorient 32, 59-79.

Conard, N., Ghasidian, E., 2011. The Rostamian Cultural group and the taxonomy of the Upper Paleolithic in Iran, in: Conard, N.J., Drechsler, P., Morales, A. (Eds.), Between Sand and See. Kerns Verlag, Thubingen.

Conard, N., Ghasidian, E., Heydari-Guran, S., 2009. The Open-air Late Paleolithic site of Bardia and the Paleolithic Occupation of the Qaleh Gusheh Sand Dunes, Esfahan Province, Iran, in: M., O., F., B., J., J. (Eds.), Iran Paelolithic. International BAR Series, Lisbon, pp. 141-154.

Coon, C.S. The Seven Caves. New York: Knopf (1957).

Coon, C.S., 1951. Cave Explorations in Iran: 1949. University Museum, Pennsylvania.

Darabi, H, et al. 2012. Palaeolithic occupation of the Mehran Plain, in Southwestern Iran. Documenta Praehistorica. XXXIX: 443-451.

Dashtizadeh, A., 2010. Preliminary report on the discovery of a Late Lower Paleolithic and Middle Paleolithic site on the Island of Qeshm. Persian Gulf: Iranian Archaeology 1, 15-20.

Davoudi, D., Bazgir, B., Abbasnejad, R., Barsky, D., Ollé, A., Otte, M., 2015. The Lower Paleolithic of Iran: Probing New Finds from Mar Gwergalan Cave (Holeylan, Central Zagros). Archaeology, Ethnology and Anthropology of Eurasia 43, 3-15.

Dibble, H., Holdaway, S., 1993. The Middle Paleolithic of Warwasi Rockshelter, in: Olszewski, D., Dibble, H.L. (Eds.), The Paleolithic Prehistory of the Zagros-Taurus. The University Museum Press, University of Pennsylvania, pp. 75-99.

Dibble, H.L., 1984. The mousterian industry from Bisitun cave (Iran). Paléorient, 23-34.
Djamali, M., Biglari, F., Abdi, K., Andrieu-Ponel, V., de Beaulieu, J.-L., Mashkour, M., Ponel, P., 2011. Pollen analysis of coprolites from a late Pleistocene–Holocene cave deposit (Wezmeh Cave, west Iran): insights into the late Pleistocene and late Holocene vegetation and flora of the central Zagros Mountains. Journal of Archaeological Science 38, 3394-3401.
Ghasidian, E, et al. 2009. Late Paleolithic Cultural Traditions in the Basht Region of the Southern Zagros of Iran. In Iran Paleolithic, edited by M. Otte, F. Biglari, and J. Jaubert. Proceedings of the XV World Congress (Lisbon, 4-9 September 2006): 125-140.
Ghasidian, E., 2014. The Early Upper Paleolithic Occupation at Ghār-e Boof Cave A Reconstruction of Cultural Tradition in the Southern Zagros Mountains of Iran. Kerns Verlag, Thubingen.
Ghasidian, E., Heydari-Guran, S., Azadi, A., Conard, N., 2009. Late Paleolithic cultural traditions in the Basht Region of the Southern Zagros of Iran, in: Otte, M., Biglari, F., Jaubert, J. (Eds.), International Union for Prehistoric and Protohistoric Science. BAR International Series, Lisbon.
Hariryan, H., Saman Heydari-Guran , Abbas Motarjem & Elham Ghasidian (2021): New Evidence of a Late Pleistocene Occupation on the Southern Slopes of the Alborz Mountains, Lithic Technology, DOI: 10.1080/01977261.2020.1860350.
Heydari, M., Guérin, G., Kreutzer, S., Jamet, G., Kharazian, M.A., Hashemi, M., Nasab, H.V., Berillon, G., 2020. Do Bayesian methods lead to more precise chronologies? 'BayLum' and a first OSL-based chronology for the Palaeolithic open-air site of Mirak (Iran). Quaternary Geochronology 59, 101082.
Heydari Guran, S., Ghasidian, E., The MUP Zagros Project: tracking the Middle Upper Palaeolithic transition in the Kermanshah region, west-central Zagros. Iran. Antiquity91, 355 (2017).
Heydari-Guran, S. & Ghasidian, E. Late Pleistocene hominin settlement patterns and population dynamics in the Zagros Mountains: Kermanshah region. Archaeological Research in Asia21, 100161 (2020).
Heydari-Guran, S. Palaeolithic Landscapes of Iran. BAR International Series, 2568 (2014), (a).
Heydari-Guran, S., Ghasidian, E., and N. Conard. 2014. Middle Paleolithic Settlements on the Iranian Central Plateau. In: Settlement Dynamics of the Middle Paleolithic and Middle Stone Age, Vol. IV, N. Conard, A. Delagnes (eds.). Pp. 171-203. Tübingen Publication in Prehistory.
Heydari-Guran, S., Katerina Douka, Thomas Higham, Susanne C. Münzel et al., Early Upper Palaeolithic occupation at Gelimgoush cave, Kermanshah; West-Central Zagros mountains of Iran, Journal of Archaeological Science: Reports, Volume 38, 2021.
Hole, F., Flannery, K.V., 1967. The prehistory of southwestern Iran: a preliminary report, Proceedings of the Prehistoric Society. Cambridge University Press, pp. 147-206.
Hume, G.W., 1976. The Ladizian: An Industry of the Asian Chopper-Chopping Tool Complex in Iranian Baluchistan. Dorrance.
Ikeda, J., 1979. Preliminary report of an archaeological survey in Arsanjan area, Fars Province, Iran, 1977. Kyoto University, Kyoto.
Jamialahmadi, M., Vahdati Nasab, H., Fazeli Nashli, H., 2008. Kashafrud Revisited, Discovery of New Paleolithic sites in Northeastern of Iran. Antiquity 82.
Jaubert, J., Biglari, F., Bordes, J.-G., Bruxelles, L., Mourre, V., Shidrang, S., 2006. The Paleolithic of Iran: Report of 2004 Iranian-French Joint Mission. Archaeological Reports 3, 1-12.
Jaubert, J., Biglari, F., Bordes, J.-G., Bruxelles, L., Mourre, V., Shidrang, S., 2006. The Paleolithic of Iran: Report of 2004 Iranian-French Joint Mission. Archaeological Reports 3, 1-12.

Jaubert, J., Biglari, F., Mourre, V., Bruxelles, L., Bordes, J.-G., Shidrang, S., Naderi, R., Alipour, S., Mashkour, M., Mallye, J.-B., Quinif, Y., Rendu, W., Laroulandie, V., 2009. The Middle Paleolithic occupation of Mar Tarik, a new Zagros Mousterian site, in: Otte, M., Biglari, F., Jaubert, J. (Eds.), International Union for Prehistoric and Protohistoric Science, Lisbon.

Lindly, J.M., 2005. The Zagros Mousterian: a regional perspective. Arizona State University, Tempe.

Mashkour, M., Monchot, H., Trinkaus, E., Reyss, J.-L., Biglari, F., Bailon, S., Heydari, S., Abdi, K., 2009. Carnivores and their prey in the Wezmeh Cave (Kermanshah, Iran): a Late Pleistocene refuge in the Zagros. International Journal of Osteoarchaeology 19, 678-694.

McBurney, C., 1970. Paleolithic Excavations in the Zagros Area. Iran 8, 185-186.

McBurney, C.B.M., 1964. Preliminary Report on Stone Age Reconnaissance in north-eastern Iran. Proceedings of the Prehistoric Society 30, 382-399.

McCormack, J., Bontognali, T. R. R., Immenhauser, A., & Kwiecien, O. (2018). Controls on Cyclic Formation of Quaternary Early Diagenetic Dolomite. Geophysical Research Letters, 45(8), 3625-3634. doi:https://doi.org/10.1002/2018GL077344.

Mortensen, P., 1993. Paleolithic and Epipaleolithic Sites in the Hulailan Valley, Northern Luristan, in: Olszewsky, D.I.a.D., H. L. (Ed.), The Paleolithic Prehistory of the Zagros-Taurus. University of Pennsylvania, Philadelphia, pp. 159-187.

Mosapour Negari, F., Zarei, S., and M. Gorgi. 2020. Revisiting the Panj Angosht Paleolithic Open- air Site, Khash, Iranian Baluchestan: Preliminary Report of the First Phase.Proceedings of the first international virtual conference on archeology of Iran and neighboring regions

Nateqi, A., Hashemi, M., Vahdati Nasab, H., et al. 2021. Quaternary Deposits and the Paleolithic Sites on the Northern Edge of Iranian Central Desert: Introduction of the Newly-found Paleolithic Sites of Shour-e Qazi and Sar-Darreh. IRQUA XX

Nikzad, M., Sediqian, H., and E. Ghasemi. 2015. New evidence of Palaeolithic activity from South Khorasan, eastern Iran. Antiquity (Project Gallery) 347.

Olszewski, D.I., 1993. The late Baradostian occupation at Warwasi rockshelter, Iran. The Paleolithic Prehistory of the Zagros-Taurus, 184-206.

Otte, M., Shidrang, S., Zwyns, N., Flas, D., 2011. New radiocarbon dates for the Zagros Aurignacian from Yafteh cave, Iran. Journal of human evolution 61, 340-346.

Parsons, B., Wright, T., Rowe, P., Andrews, J., Jackson, J., Walker, R., . . . Engdahl, E. R. (2006). The 1994 Sefidabeh (eastern Iran) earthquakes revisited: new evidence from satellite radar interferometry and carbonate dating about the growth of an active fold above a blind thrust fault. Geophysical Journal International, 164(1), 202-217. doi:10.1111/j.1365-246X.2005.02655.x.

Piperno, M. Jahrom, a Middle Paleolithic Site in Fars, Iran. East andWest, 22: 183-97 (1972).

Piperno, M., 1974. Upper Palaeolithic Caves in Southern Iran Preliminary Report. East and west 24, 9-13.

Rosenberg, M. Paleolithic settlement pattern in the Marv Dasht, Fars Province, Iran. Unpublished Ph.D. dissertation. University of Pennsylvania. USA (1988).

Rosenberg, M., 1985. Report on the 1978 Sondage at Eshkaft-e Gavi. Iran 23, 51-62.

Rosenberg, M., 2003. The Epipaleolithic in Marv Dasht in: Miller, N.F., Abdi, K. (Eds.), Yeki Bud, Yeki Nabud, Essays on the Archaeology of Iran. The Costen Institute of Archaeology, University of California, Los Angeles, pp. 98-108.

Roustaei, K., 2010. Discovery of Middle Palaeolithic occupation at high altitude in the Zagros Mountains, Iran. Antiquity 84.

Roustaei, K., Biglari, F., Heydari, S., Vahdatinasab, H., 2002. New research on the Palaeolithic of Lurestan, West Central Iran. Antiquity 76, 19-20.

Roustaei, K., Vahdati Nasab, H., Biglari, F., Heydari-Guran, S., Clark, G., M. Lindly, J., 2004. Recent Paleolithic Surveys in Luristan. Current Anthropology 45, 692-707.

Roustaei, K., Vahdati Nasab, H., Biglari, F., Heydari-Guran, S., Clark, G., M. Lindly, J., 2004. Recent Paleolithic Surveys in Luristan. Current Anthropology 45, 692-707.

Sadek-Kooros, H., 1976. Early Hominid Traces in East Azarbaijan, in: Bagherzadeh, F. (Ed.), Proceedings of the IVth Annual Symposium on Archaeological Research in Iran. Iranian Center for Archaeological Research, Tehran.

Sadraei, A. and B. Anani. 2018. Kalat-e Shour, Sarayan, evidence of the presence of Pleistocene Population in the Eastern Iranian plateau. L'Anthropologie 122(5): 722-736.

Sadraei, A., M. Fallah Mehneh, H. Saburi, O. Garazhian, M. Azar. 2017. New Evidence of Middle Paleolithic Period in Sabzevar Plain, Northeast of Iran. Archaeology 5(1): 1-6

Sadraei, A., (2019a). Tabarak Open-Air Site, Possible Evidence from the Early Paleolithic Period on the Dry River Bed of Atrak, Quchan Plain, Iran. Indian Journal of Archaeology.

Sadraei, A., Mehneh, M. F., Sheikh, M., Anani, B., & Minaei, Z. H. (2019b). Kaftar Kouh of Ferdous, New Evidence of Paleolithic Population in Southern Khorasan, Iran. Advances in Anthropology, 9, 111-123.

Sadraei, A., Mehneh, M.F., Toghraei, M., Anani, B., & Azar, M. 2018. The Probable Lower Paleolithic Evidence from Darongar River, Dargaz, North Khorasan, Iran. Archaeology, 6, 6-11.

Shidrang, S. 2009. A Typo-Technological Study of an Upper Paleolithic Collection From Sefid-Ab, Central Iran. In Iran Paleolithic, edited by M. Otte, F. Biglari, and J. Jaubert. Proceedings of the XV World Congress (Lisbon, 4-9 September 2006): 47-56.

doi:https://doi.org/10.1016/j.quascirev.2015.07.006.

Shoaee, M.J., et al…, Initial Upper Paleolithic Entry into the Zagros Mountains, Nature (in prep), 2023.

Skinner, J. H. 1965. The Flake industries of southwest Asia: a typological study, Unpublished Ph.D. dissertation, Columbia University.

Torkamandi, S., and M. Khodabakhshi Parizi. 2018. Paleolithic survey in the Pariz region, Southern Iran. L'Anthropologie 122(5): 709-721.

Trinkaus, E., Biglari, F., Mashkour, M., Monchot, H., Reyss, J.-L., Rougier, H., Heydari, S., Abdi, K., 2008. Late Pleistocene human remains from Wezmeh Cave, western Iran. American Journal of Physical Anthropology 135, 371-378.

Tsuneki, A., Mirzaii, A., Hourshid, S., 2012. The Arsanjan Project 2011-2012, The 11th Annual Symposium of Iranian archaeology. Iranian Center for Archaeological Center, National Museum of Iran, Tehran.

Vahdati Nasab, et al. 2010a. Palaeolithic Ladiz revisited: a reassessment, of the Ladizian lithic industry, Baluchestan, Iran; Antiquity. Volume 84, issue 324. project gallery.

Vahdati Nasab, H, and G. A. Clark. 2014. The Upper Paleolithic of the Iranian Central Desert: the Delazian Sites, Semnan Province – a Case Study. Archaologische Mitteilungen aus Iran und Turan (AMIT).

Vahdati Nasab, H, and Z. Feiz. 2014. Preliminary Report of the surface survey in the West Semnan Area. Proceedings of the 12th congress on iranian archaeology 465-468.

Vahdati Nasab, H, et al. 2008. Helak, a Paleolithic Cave Complex Featuring Rock art along the Northern Shore of Parishan Lake, Fars province, Iran.Nameh Pajouheshgah, International volume, 22 and 23: 91-96.

Vahdati Nasab, H, et al. 2009. Paleolithic Levalloisian Assemblages from Boeen Zahra in the Qazvin Plain (Iran). Antiquity. Volume 83, issue 320. project gallery

Vahdati Nasab, H., Berillon, G., Jamet, G., Hashemi, M., et al. 2019. The open-air Paleolithic site of Mirak, northern edge of the Iranian Central Desert (Semnan, Iran): Evidence of repeated human occupations during the late Pleistocene. Comptes Rendus Palevol 18, 465-478.

Vahdati Nasab, H., Hajjami, S., and M. Mortazavi. 2010. Palaeolithic Ladiz revisited: a reassessment of theLadizian lithic industry, Baluchestan, Iran. Antiquity (Project Gallery) 084(324).

Vahdati Nasab, H., Hashemi, M., 2016. Playas and Middle Paleolithic settlement of the Iranian Central Desert: The discovery of the Chah-e Jam Middle Paleolithic site. Quaternary International 408, 140-152.

Vahdati Nasab, H., Roustaei, K., Ghamari Fatideh, M., Shojaeefar, F., & Hashemi Sarvandi, M. (2017). The first evidence for Late Pleistocene hominin populations on the southern Caspian Sea coast. Antiquity, 91(355), E1.

Yousefi, M., Heydari-Guran, S., Kafash, A. et al. Species distribution models advance our knowledge of the Neanderthals' paleoecology on the Iranian Plateau. Sci Rep 10, 14248 (2020).

Zeidi, M., Barfi, C., & Zare, S., International research journal of modernization in engineering technology and science, Discovery of new Palaeolithic localities in the southern margins of Lut desert, south-eastern Iran.
