# Supplementary material for: Defining paleoclimatic routes and opportunities for hominin dispersals across Iran
Source: PLoS One. 2023 Mar 1;18(3):e0281872. doi: 10.1371/journal.pone.0281872 (PMC9977010; doi:10.1371/journal.pone.0281872)
Supplement: S2 Table — (PDF) [file pone.0281872.s002.pdf]

SI 2 – PALAEOENVIRONMENTAL PROXIES USED IN THIS STUDY

Site name	Lat	Long	Reference	Fig 3 site #	MIS humidity
Urmia	37.76506	45.34065	Djamali et al., 2008	1	5, 7
Van	38.65532	42.93159	Litt et al., 2014; McCormack et al., 2018	2	5, 3
Zeribar	35.53333	46.13333	Van Zeist et al., 1963	3	3?
Mirabad	33.08336	47.71669	Stevens et al., 2006	4	3?
Qale'Kord	35.79722	48.85722	Mehterian et al., 2017	5	5,1
Zarand	35.34927	50.45195	Djamali et al., 2006	6	U
Toshan	36.81694	54.42361	Kehl et al., 2021; Vlaminck et al., 2016	7	5,3
Agh Band	37.62167	55.16083	Lauer et al., 2017	8	5,3
Neka-Abelou	36.64528	53.32083	Kehl et al., 2021	9	5,3
Kalat-e Naderi	37.00222	59.72806	Karimi et al., 2011	10	5
Pir Ghar	35.23	57.42	Carolin et al., 2019	11	5
Kopet Dagh	37.615	58.07167	Shabanian et al., 2009	12	Mid Pleist & 5
Neyshabour	36.295	58.65167	Walker & Fattahi 2011, Hollingsworth et al.,2010	13	3/4
Kashmar (UchPalang)	35.2827	58.57317	Walker & Fattahi 2011, Fattahi et al., 2007	14	3
Nimbluk basin	34.03413	58.83403	Fattahi & Walker 2015	15	1
Alborz Neor	37.62694	48.55528	Sharifi et al., 2015	16	1
East Ishfahan	32.40003	52.06669	Bayat et al., 2018	17	5,11
Sefidabeh	30.97175	60.52798	Parsons et al.,2006	18	5,1
Gavkhoni	32.16669	58.76669	Jones et al., 2014	19	U
Pishamak	26.398	61.43	Kober et al., 2013	20	5,3
Rask	26.19	61.459	Kober et al., 2013	21	7
Ghasr-Ghand	26.198	61.084	Kober et al., 2013	22	6,3
Hoshomb	25.824	60.692	Kober et al., 2013	23	5,3,1
Minab	27.5	57.25	Walker & Fattahi 2011, Regard et al.,2006	24	3,2,1
Jazmurian	27.61778	58.58806	Vaezi et al., 2019	25	1
Sabzevar	36.22205	57.52587	Walker & Fattahi 2011, Fattahi et al., 2006	26	1
Kashmar (SheshTaraz)	35.29675	58.16282	Walker & Fattahi 2011, Fattahi et al., 2007	27	1,2
Anar	31.19988	55.15228	Walker & Fattahi 2011, Le Dorz et al., 2009	28	1
Bam	29.01195	58.39972	Walker & Fattahi 2011	29	1
South Golbaf	29.78865	57.77455	Walker & Fattahi 2011	30	1

Proxy data references not cited in main text are listed below (all others can be found in manuscript)

Fattahi, M., Walker, R., Hollingsworth, J., Bahroudi, A., Nazari, H., Talebian, M., Armitage, S., Stokes, S., 2006. Holocene slip-rate on the Sabzevar thrust fault, NE Iran, determined using optically stimulated luminescence (OSL). Earth and Planetary Science Letters 245, 673e684.

Fattahi, M., Walker, R.T., Khatib, M.M., Dolati, A., Bahroudi, A., 2007. Slip-rate estimate and past earthquakes on the Doruneh fault, eastern Iran. Geophysical Journal International 168, 691e709

Hollingsworth, J., Fattahi, M., Walker, R., Talebian, M., Bahroudi, A., Bolourchi, M.J., Jackson, J., Copley, A., 2010. Oroclinal bending, distributed thrust and strike-slip faulting, and the accommodation of Arabia-Eurasia convergence in NE Iran since the Oligocene. Geophysical Journal International.

Regard, V., Bellier, O., Braucher, R., Gasse, F., Bourles, D., Mercier, J., Thomas, J.-C., Abbassi, M.R., Shabanian, E., Soleymani, Sh, 2006. 10Be dating of alluvial deposits from southeastern Iran (the Hormoz Strait area). Palaeogeography, Plaeoclimatology, Palaeoecology 242, 36e53

Le Dortz, K., Meyer, B., Sebrier, M., Nazari, H., Braucher, R., Fattahi, M., Benedetti, L., Foroutan, M., Siame, L., Bourles, D., Talebian, M., Bateman, M.D., Ghoraishi, M., 2009. Holocene right-slip rate determined by cosmogenic and OSL dating on the Anar fault, Central Iran. Geophysical Journal International 179, 700e710.
